# Integrative multi-omics analysis identifies a PTM-related immune signature and IRF9 as a driver in ccRCC

**DOI:** 10.3389/fimmu.2025.1707375

**Published:** 2025-12-01

**Authors:** Zixiang Li, Xun Li, Simeng Hu, Shan Jiang, Junqi Wang

**Affiliations:** 1 Department of Urology, The Affiliated Hospital of Xuzhou Medical University, Xuzhou, China; 2 Emergency Center of the Affiliated Hospital of Xuzhou Medical University , Xuzhou, China; 3 Department of General Surgery, The Affiliated Hospital of Xuzhou Medical University, Xuzhou, China

**Keywords:** ccRCC, post-translational modifications (PTMs), tumor microenvironment, immunotherapy, single-cell RNA sequencing, IRF9

## Abstract

**Background:**

Clear cell renal cell carcinoma (ccRCC) exhibits marked heterogeneity and variable benefit from immune checkpoint inhibitors (ICIs). Post-translational modifications (PTMs) regulate immune signaling and tumor behavior, yet PTM-informed biomarkers for ccRCC remain underexplored.

**Methods:**

We intersected immune-related genes, PTM-related genes, and differentially expressed genes in TCGA-KIRC to derive candidates and built a prognostic model across TCGA and E-MTAB-1980 using multiple algorithms, selecting a random survival forest-based post-translational modification-related signature (PTMRS) with the best performance. Prognostic value and independence were evaluated by time-dependent ROC, Kaplan-Meier, and multivariate Cox analyses. Tumor immune context was profiled by immune infiltration scores, immune checkpoints, and TIDE to infer dysfunction/exclusion and ICI response. Genomic features (driver mutations, pathway alterations, tumor mutational burden) and an external ICI cohort (IMvigor210) were analyzed. Single-cell RNA-seq with CellChat, trajectory, and UCell assessed cell-cell communication and PTMRS distribution across immune subsets. Experimental validation included IHC and qPCR of IRF9, loss- and gain-of-function assays (Transwell, wound healing, CCK-8, colony, EdU), and molecular dynamics to explore IRF9 compound binding.

**Results:**

We established a five-gene PTMRS that robustly stratified prognosis in training, testing, and external cohorts and remained an independent predictor. High-PTMRS tumors displayed immunosuppressive features, including greater infiltration of Tregs/MDSCs/macrophages, higher expression of immunosuppressive checkpoints, and elevated TIDE scores with lower predicted ICI responsiveness. High PTMRS associated with alterations in oncogenic pathways and higher TMB. In IMvigor210, high PTMRS linked to inferior outcomes and non-response. Single-cell analyses showed PTMRS enrichment in Tregs and exhausted CD8^+^ T cells and dense immune communication networks. Among PTMRS genes, IRF9 was upregulated in ccRCC tissues and cell lines. Knockdown of IRF9 curtailed invasion, migration, and proliferation, whereas overexpression enhanced these phenotypes.

**Conclusion:**

PTMRS is a PTM-informed immune signature that reflects an immunosuppressive tumor microenvironment, improves prognostic stratification, and indicates ICI benefit in ccRCC. Experimental data pinpoint IRF9 as a functional driver and potential therapeutic target within this PTM-immunity axis.

## Introduction

Clear cell renal cell carcinoma (ccRCC) is the most common histologic subtype of renal cell carcinoma, comprising roughly 70–80% of cases and accounting for most kidney cancer–related deaths worldwide (1, 2). Despite advances with targeted agents and immune checkpoint inhibitors (ICIs), a substantial proportion of patients ultimately relapse or develop resistance (3, 4). The complex tumor-immune crosstalk therefore underscores the need for new prognostic markers and therapeutic strategies (5).

Post-translational modifications (PTMs) are fundamental biochemical events that fine-tune protein stability, localization, and activity (6). Among these PTMs, ubiquitination represents one of the most versatile and extensively studied regulatory mechanisms, governing protein turnover, signaling, and immune responses across physiological and pathological contexts (7). Via phosphorylation, ubiquitination, acetylation, methylation, SUMOylation and other mechanisms, PTMs coordinate signaling networks governing proliferation, apoptosis, DNA repair, and immune homeostasis (8, 9). Disruption of PTM processes is increasingly recognized as a hallmark of cancer, driving malignant transformation, treatment resistance, and remodeling of the tumor microenvironment (TME) (10).

Importantly, PTMs are emerging as key regulators of immune responses within the TME. Aberrant PTM events can modulate antigen presentation, cytokine signaling, immune checkpoint expression, and T-cell activation, thereby facilitating immune evasion (11, 12). A recent comprehensive review underscored the central role of PTM-dependent immune regulation across multiple tumor types, further emphasizing the relevance of PTM–immune crosstalk in cancer biology (13). For instance, ubiquitination and acetylation of immune-regulatory proteins have been shown to influence PD-1/PD-L1 signaling, while phosphorylation-dependent pathways modulate T-cell receptor (TCR) activity (14, 15). Although PTM dysregulation has been extensively characterized in other malignancies such as hepatocellular, lung, and colorectal cancers, research on PTM-mediated regulation in ccRCC remains relatively limited. Similar mechanisms have been described for non-coding RNA-mediated epigenetic modulation of immune signaling, underscoring the complexity of PTM-epigenetic interactions in tumor immunity (16). This may be due to the distinct metabolic and immune landscape of ccRCC, which complicates direct extrapolation from other tumor contexts. Only a few recent studies have begun to address PTM-related mechanisms in renal cancer, underscoring the need for systematic investigation (17). These findings suggest that PTM-related genes may serve as crucial molecular determinants linking tumor biology with antitumor immunity, yet their prognostic and immunological implications in ccRCC remain poorly understood.

Given this gap, systematic characterization of PTM-associated immune signatures could provide novel insights into tumor progression and immune escape in ccRCC. Moreover, integrating PTM-related immune genes into prognostic modeling may enable more accurate risk stratification and offer potential guidance for immunotherapy. Therefore, in the present study, we aimed to comprehensively investigate PTM-associated immune genes in ccRCC, construct a prognostic signature, and explore their relationship with the immune landscape, with the goal of identifying potential therapeutic targets and improving patient management.

## Methods

### Data acquisition and preprocessing

RNA-seq profiles and matched clinical annotations for patients with ccRCC were sourced from The Cancer Genome Atlas (TCGA). An external validation cohort (E-MTAB-1980) was downloaded from the ArrayExpress repository. Immune-related genes were drawn from the ImmPort database, and PTM-associated genes were collated from the published report (18). Differentially expressed genes (DEGs) were identified using |log2FC| > 1 and Benjamini-Hochberg-adjusted p < 0.05. The single-cell data were obtained from GEO (accession: GSE159115), and the spatial transcriptomics data were obtained from SpatialTME (https://www.spatialtme.yelab.site/).

### Construction of prognostic model

A multilayer perceptron neural network was first constructed as a benchmarking model to assess the intrinsic predictive potential of PTM-related genes prior to selection of the optimal machine-learning algorithm for post-translational modification–related risk score (PTMRS) construction. Candidate PTM-immune genes were defined as the overlap among the immune-related genes, PTM-related genes, and DEGs in TCGA. Genes associated with overall survival were first screened by univariable Cox regression. Multiple machine learning algorithms were applied to construct prognostic models across the training and validation cohorts. Model performance was evaluated using concordance index (C-index) and time-dependent receiver operating characteristic (ROC) curves, and the Random Survival Forest (RSF), which achieved the highest prognostic accuracy, was selected to establish the PTM-related risk score (PTMRS). Patients were stratified into high- and low-risk groups according to the median PTMRS. For instance, by locating each patient’s values for age, stage, grade, and PTMRS on the respective axes, the corresponding points are summed to obtain a total score, which is then projected onto the survival scale to determine individualized survival probability.

### Immune landscape analysis

The TME was profiled using the single-sample gene set enrichment analysis (ssGSEA) to quantify immune-cell infiltration. Immune checkpoint expression and immune-related signatures were compared across PTMRS-defined groups. Predicted responsiveness to immune checkpoint blockade was inferred with the TIDE framework. Associations between PTMRS and indices of immunosuppression were then systematically examined.

### Statistical analysis

All analyses were carried out in R (version 4.2.1). Between-group differences were assessed with Student’s t test or the Wilcoxon rank-sum test, as appropriate. Two-sided p values < 0.05 were considered statistically significant. Detailed experimental procedures are provided in **Supplementary Table 1**.

## Results

### Construction and performance evaluation of a neural network model based on post-translational modification-related genes

As shown in **Supplementary Figure S1**, we established a multilayer perceptron neural network using post-translational modification-related genes (PTMRGs). After 100 training epochs, the model achieved stable convergence with an accuracy of 0.98 and a loss of 0.05 (**Supplementary Figure S1A**). In the training cohort, the model demonstrated nearly perfect classification performance (sensitivity 0.997, specificity 1.0, accuracy 0.998), with an area under the curve (AUC) of 1.000 (**Supplementary Figures S1B–D**). In the independent testing cohort, the model retained high predictive power, achieving a sensitivity of 0.975, specificity of 0.9, accuracy of 0.956, and an AUC of 0.942 (**Supplementary Figures S1E–G**). External validation in the GSE40435 dataset further confirmed its generalizability, with a sensitivity of 1.0 and an AUC of 0.906, although specificity was reduced (0.545), resulting in a moderate overall accuracy of 0.772 (**Supplementary Figures S1H–J**). Collectively, these results indicate that the neural network model based on post-translational modification-related genes exhibits high accuracy and robustness across training and testing datasets and maintains reliable predictive ability in external validation, supporting its potential utility in immunological research and clinical applications.

### Identification of prognostic PTM-related immune genes and construction of the prognostic model

**Figure 1:**
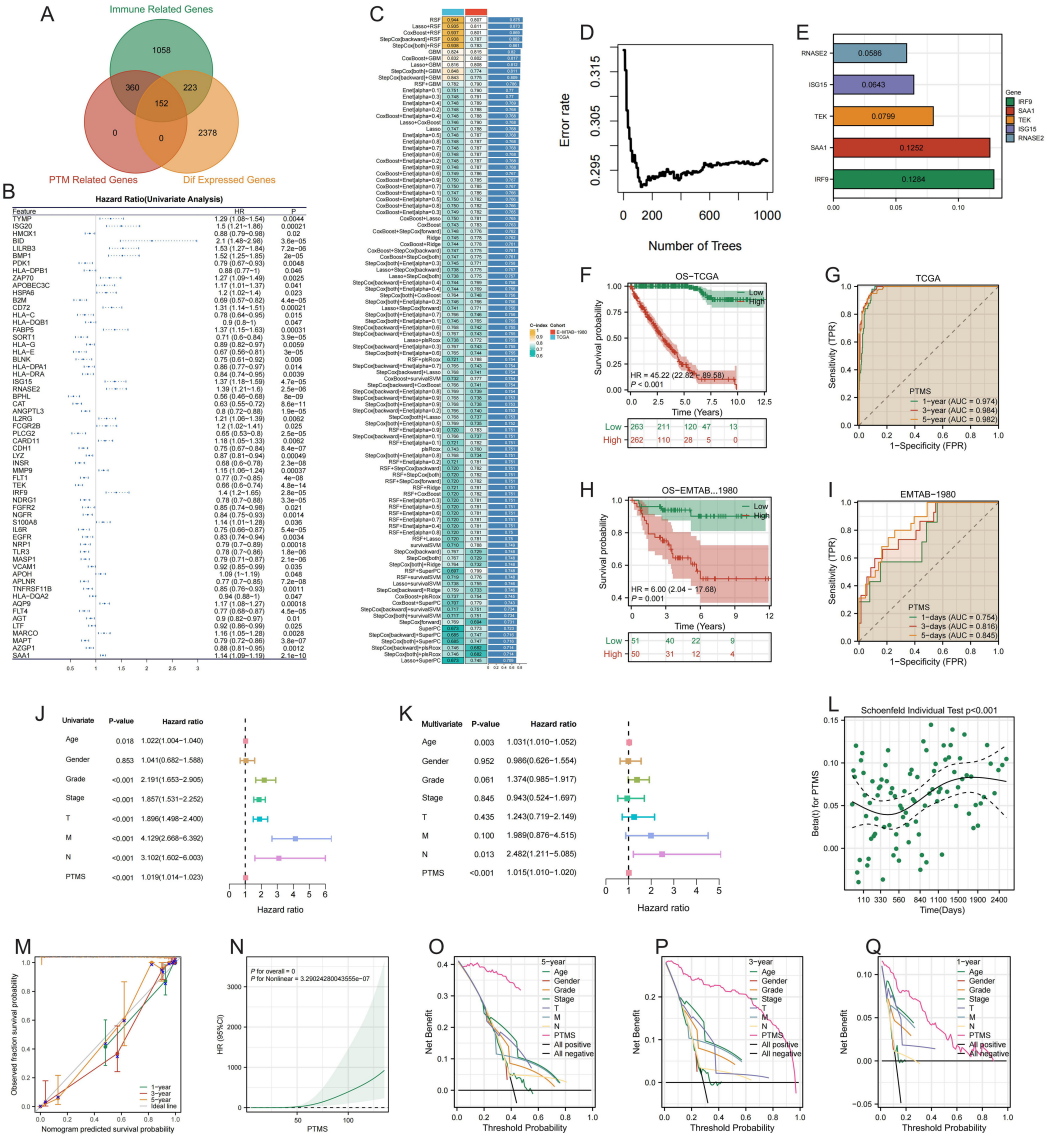
Prognostic model based on PTM-related immune genes. **(A)** Intersection of immune-, PTM-related, and differentially expressed genes. **(B)** Univariate Cox analysis identifying prognostic genes. **(C–E)** Model construction using multiple algorithms, with RSF selected as optimal. **(F–I)** Kaplan-Meier (KM) and ROC analyses in TCGA and E-MTAB-1980. **(J, K)** Cox regression confirming model independence. **(L)** Correlation of risk score with survival. **(M–Q)** Calibration and decision curve analyses validating predictive accuracy and clinical benefit. Multiple panels controlling for multiple features were BH-FDR corrected.

Immune-related genes, PTM-related genes, and DEGs in TCGA were intersected, yielding 152 overlapping candidates (**Figure 1A**). The complete list of overlapping PTM-immune-DEGs is provided in **Supplementary Table 2** for reference. Univariable Cox screening then identified genes significantly associated with overall survival (**Figure 1B**). These prognostic genes were subsequently modeled using multiple algorithms across two independent datasets, and the RSF method, which achieved the highest performance index, was ultimately selected for model construction (**Figures 1C–E**). The RSF-based prognostic risk score effectively stratified patients, with high-PTMRS group exhibiting markedly poorer overall survival in both the TCGA cohort (**Figure 1F**) and the E-MTAB-1980 validation cohort (**Figure 1H**). ROC analyses further demonstrated robust predictive performance, with AUCs of 0.717 and 0.653 at 5 years in TCGA and E-MTAB-1980, respectively (**Figures 1G, I**). Univariable and multivariable Cox analyses confirmed that the PTMRS was an independent prognostic factor beyond conventional clinical variables (**Figures 1J, K**). The PTMRS showed a significant association with survival outcomes (**Figure 1L**), while calibration and decision curve analyses verified the predictive accuracy and clinical benefit of the integrated nomogram (**Figures 1M–Q**). Collectively, these findings demonstrate that the PTMRS possesses strong prognostic value and represents a reliable tool for individualized risk stratification in ccRCC.

### The association between PTMRS with immunosuppressive tumor microenvironment

**Figure 2:**
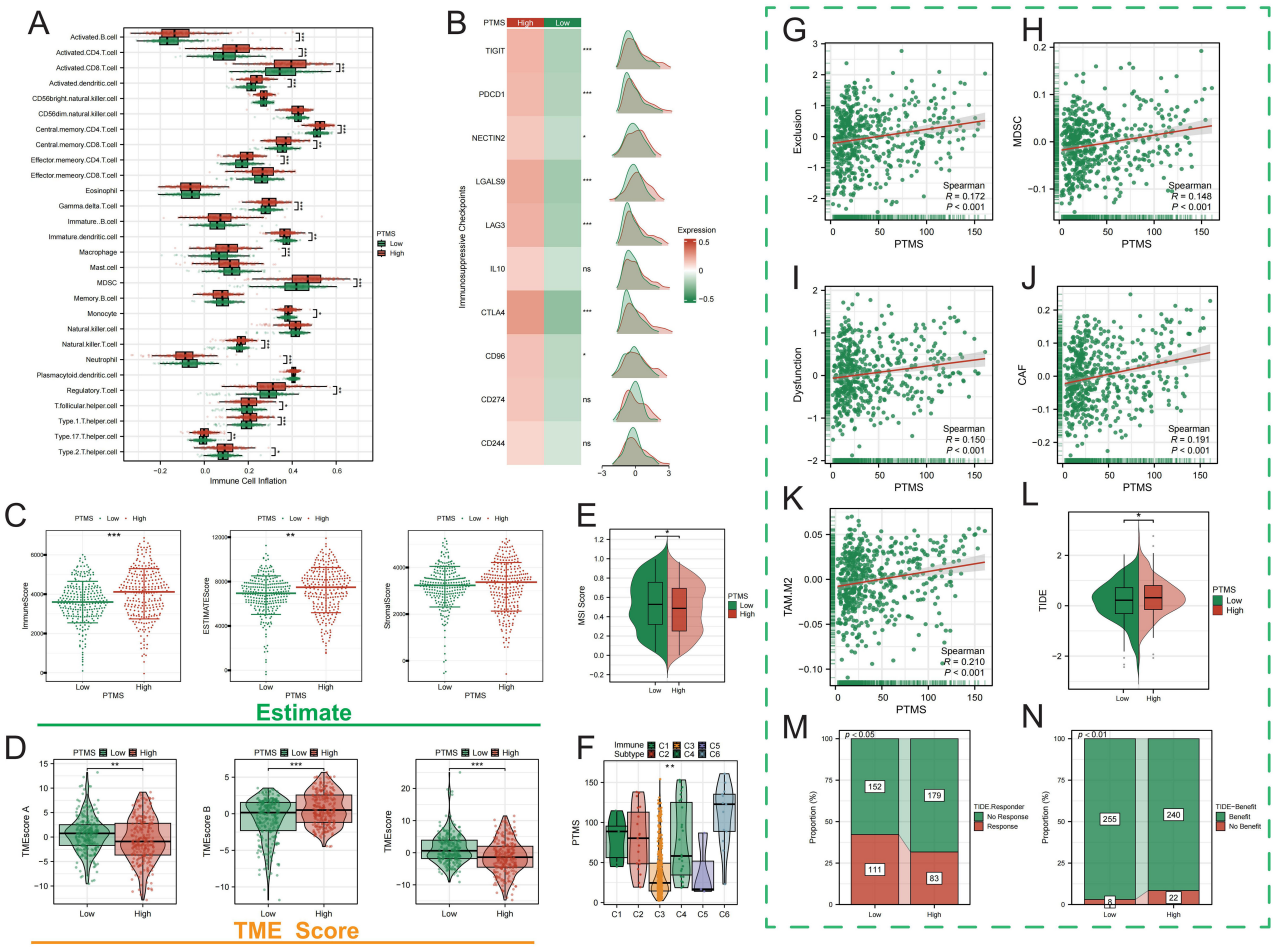
PTMRS and immunosuppressive tumor microenvironment in KIRC. **(A)** Immune cell infiltration between high- and low-PTMRS groups. **(B)** Expression of immune checkpoints (PDCD1, CTLA4, LAG3, TIGIT). **(C–E)** Stromal and immune scores in different risk groups. **(F)** Distribution of PTMRS across immune subtypes. **(G–K)** TIDE-predicted infiltration of immunosuppressive cell. **(L)** Association of PTMRS with TIDE score. **(M, N)** TIDE-predicted immunotherapy response in high- and low-PTMRS groups.

Immune infiltration analysis revealed markedly higher proportions of immunosuppressive cell populations in the high-PTMRS group, including myeloid-derived suppressor cells (MDSCs), macrophages, and regulatory T cells (Tregs) (**Figure 2A**). Patients with high PTMRS exhibited significantly elevated expression of immune checkpoints, indicating stronger inhibitory signaling (**Figure 2B**). They also showed higher stromal and immune scores (**Figures 2C–E**), consistent with a more complex TME. Distribution analysis across immune subtypes confirmed that PTMRS was enriched in immunosuppressive phenotypes (**Figure 2F**). Using the TIDE algorithm, we further evaluated the association between PTMRS and immune suppression. High PTMRS was strongly correlated with increased infiltration of immunosuppressive cells, including MDSCs, CAFs, and Tregs (**Figures 2G–K**). TIDE analysis also demonstrated that patients with high PTMRS had significantly elevated TIDE scores (**Figure 2L**), suggesting enhanced immune dysfunction and escape potential. Importantly, TIDE-predicted response analysis revealed that high-PTMRS group exhibited non-response to immune checkpoint blockade, while those with low PTMRS had a higher proportion of responders (**Figures 2M, N**). Together, these findings indicate that PTMRS is closely associated with immunosuppressive cell infiltration, immune escape, and immunotherapy resistance, highlighting its potential as a biomarker of the immunosuppressive TME in ccRCC.

### Genomic alterations, TMB, and immunotherapy response associated with PTMRS

**Figure 3:**
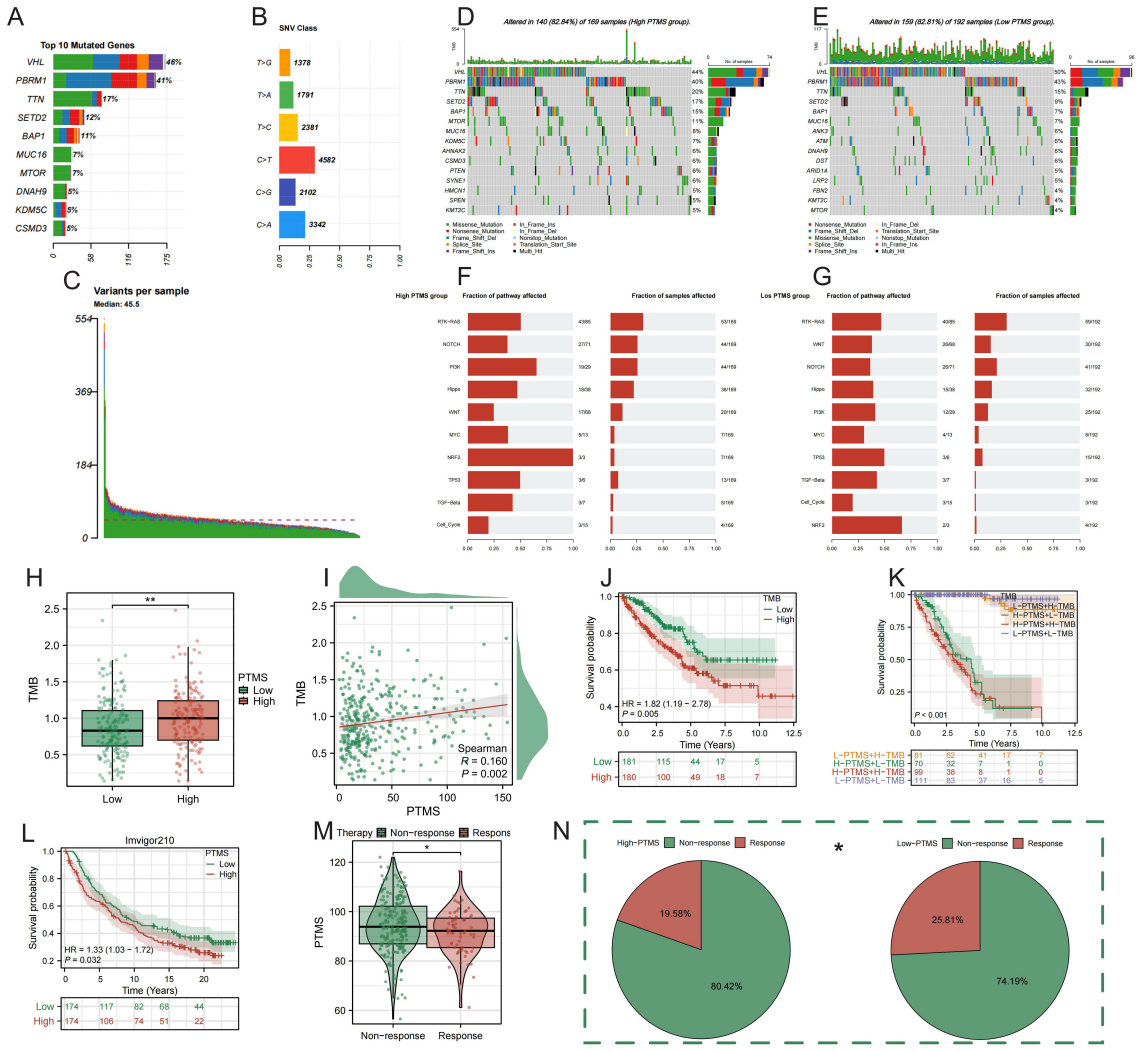
Genomic alterations, TMB, and immunotherapy response associated with PTMRS. **(A–E)** Somatic mutation landscape of high- and low-PTMRS groups. **(F, G)** Pathway alterations enriched in oncogenic signaling. **(H)** Expression of TMB in PTMRS groups. **(I)** Correlation between PTMRS and TMB. **(J, K)** Survival analysis based on PTMRS and TMB levels. **(L)** Validation of PTMRS in the IMvigor210 immunotherapy cohort. **(M, N)** Association between PTMRS and clinical response to immune checkpoint blockade.

Mutation analysis revealed frequent alterations in classical driver genes in both high- and low-PTMRS groups, including VHL, PBRM1, SETD2, and BAP1, with more than 80% of samples carrying at least one mutation (**Figures 3A–E**). Subgroup comparison of mutation frequencies revealed that VHL mutations were significantly more prevalent in low-PTMRS tumors (55.6% *vs*. 39.2%; χ² = 6.13, p = 0.013). These findings suggest distinct genomic landscapes associated with PTMRS-defined risk groups. Pathway analysis indicated that high PTMRS tumors were more likely to harbor alterations in key oncogenic pathways (**Figures 3F, G**). Tumor mutational burden (TMB) was elevated in the high PTMRS group and positively correlated with PTMRS (**Figures 3H, I**). Subgroup analyses further confirmed that elevated TMB alone correlated with inferior survival (**Figure 3J**). Furthermore, joint stratification by PTMRS and TMB demonstrated that patients with concomitantly high PTMRS and high TMB experienced the poorest prognosis, whereas those with low PTMRS and high TMB achieved the most favorable outcomes (**Figure 3K**). External validation in the IMvigor210 immunotherapy cohort revealed that the high-PTMRS group had significantly worse outcomes (**Figure 3L**). Moreover, high PTMRS patients exhibited a markedly higher rate of non-response to immune checkpoint blockade, further supporting its association with immune resistance (**Figures 3M, N**). Although IMvigor210 is derived from urothelial carcinoma, it serves as a useful reference for ICB responsiveness, as both RCC and bladder cancer share PD-1/PD-L1–related immunobiology. Still, extrapolation across cancer types should be interpreted cautiously, and validation in RCC-specific ICI cohorts is warranted once available. Together, these findings indicate that PTMRS reflects distinct mutational and pathway features, correlates with TMB, and predicts poor immunotherapy efficacy.

### Prognostic significance of PTMRS genes in ccRCC

**Figure 4:**
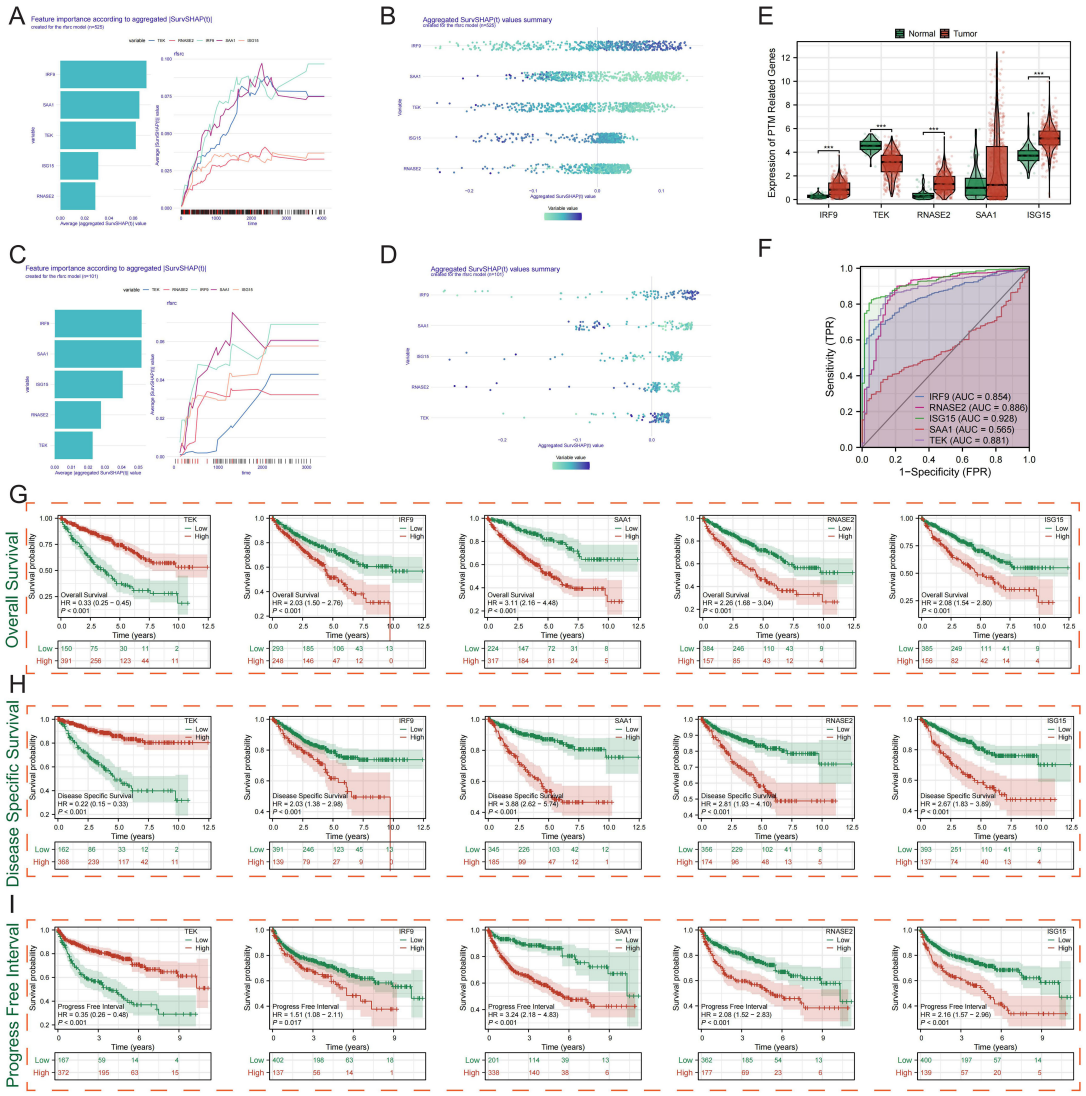
Prognostic significance of PTMRS genes in KIRC. **(A, B)** SurvSHAP(t) analysis in the TCGA cohort identifying the contribution of PTMRS genes to the model. **(C, D)** Validation of gene importance in the E-MTAB-1980 dataset. **(E)** Differential expression of PTMRS genes between tumor and normal tissues. **(F)** ROC curves showing predictive performance of PTMRS genes. **(G–I**) KM survival analyses demonstrating overall survival, disease-specific survival, and progression-free interval associated with gene expression.

Using time-dependent SHAP for survival (SurvSHAP(t)) analysis in the TCGA cohort (**Figures 4A, B**) and validated in the E-MTAB-1980 dataset (**Figures 4C, D**), IRF9, SAA1, ISG15, RNASE2, and TEK were identified as the main contributors to the PTMRS model. Expression analysis revealed significant dysregulation of these genes between tumor and normal tissues (**Figure 4E**) and ROC curves showed strong predictive ability for several genes (**Figure 4F**). Survival analyses demonstrated that high expression of IRF9, ISG15, RNASE2, and SAA1 was significantly associated with poorer overall survival, while TEK expression correlated with favorable prognosis (**Figure 4G**). Consistent patterns were observed in disease-specific survival (**Figure 4H**) and progression-free interval (**Figure 4I**), further confirming the prognostic value of these PTMRS genes. Taken together, IRF9 emerged as the most stable and clinically relevant PTMRS gene across both datasets, and was therefore selected for subsequent in-depth analysis.

### Single-cell transcriptomic analysis revealed PTMRS distribution and immune interactions in ccRCC

**Figure 5:**
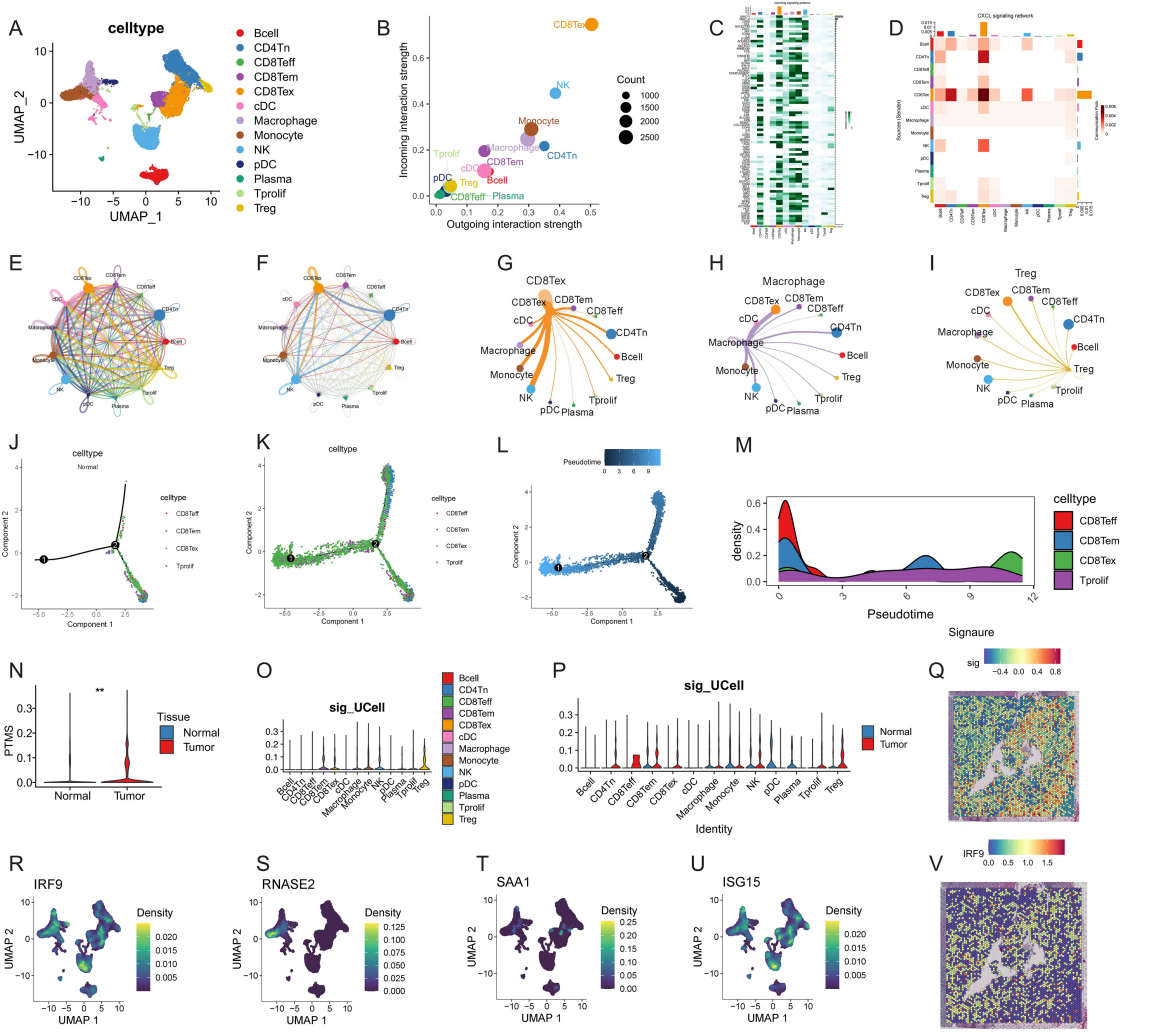
Single-cell transcriptomic analysis of PTMRS in ccRCC. **(A)** UMAP clustering of immune cell subpopulations. **(B)** CellChat analysis of interaction strengths. **(C–D)** Heatmap and pathway contribution analysis. **(E–I)** Network visualization of immune cell communication. **(J–M)** Pseudotime trajectories of CD8^+^T-cell subsets. **(N)** PTMRS scores in tumor versus normal tissues. **(O, P)** UCell analysis of PTMRS distribution. **(Q)** PTMRS signatures in paired ccRCC tumor sample. **(R–U)** Expression of PTMRS core genes (IRF9, RNASE2, SAA1, ISG15). **(V)** IRF9 expression in ccRCC tumor sample.

At the single-cell level, clustering of immune cells identified multiple subpopulations (**Figure 5A**). CellChat analysis revealed extensive intercellular communications, with CD8Teff, macrophages, and NK cells showing strong outgoing and incoming signaling strengths (**Figure 5B**). The global interaction heatmap and signaling contribution analysis further highlighted pathways such as MHC-I/II, CCL, ICAM, GALECTIN, and TNF as critical mediators of immune crosstalk (**Figures 5C, D**). Network visualization confirmed dense signaling among immune subsets, with CD8^+^ T cells, macrophages, and Tregs functioning as major hubs (**Figures 5E–I**). Trajectory inference mapped pseudotime development of T cell subsets, demonstrating transitions from effector to memory and exhausted states, as well as proliferating T cells (**Figures 5J–M**). Quantitative comparison revealed that the PTMRS was significantly higher in tumor samples (**Figure 5N**). Single-cell signature scoring method (UCell) analysis showed PTMRS enrichment in immunosuppressive subsets, particularly Tregs and exhausted CD8^+^ T cells, while distribution patterns differed between tumor and normal tissues (**Figures 5O, P**). Spatial distribution maps further confirmed that PTMRS signatures (**Figure 5Q**) and IRF9 expression (**Figure 5V**) were markedly elevated in tumor tissues. Expression mapping of PTMRS core genes revealed distinct distribution patterns at the single-cell level. Specifically, IRF9 showed high density in CD8^+^ T cells and Tregs, RNASE2 was enriched in myeloid subsets, SAA1 was broadly expressed in inflammatory clusters, and ISG15 was preferentially detected in interferon-responsive populations (**Figures 5R–U**). Collectively, these results indicate that PTMRS is heterogeneously distributed among immune subsets, preferentially enriched in immunosuppressive and exhausted phenotypes, and closely associated with IRF9 expression at the single-cell level in ccRCC.

### Molecular docking and molecular dynamics simulation of IRF9 with candidate compounds

**Figure 6:**
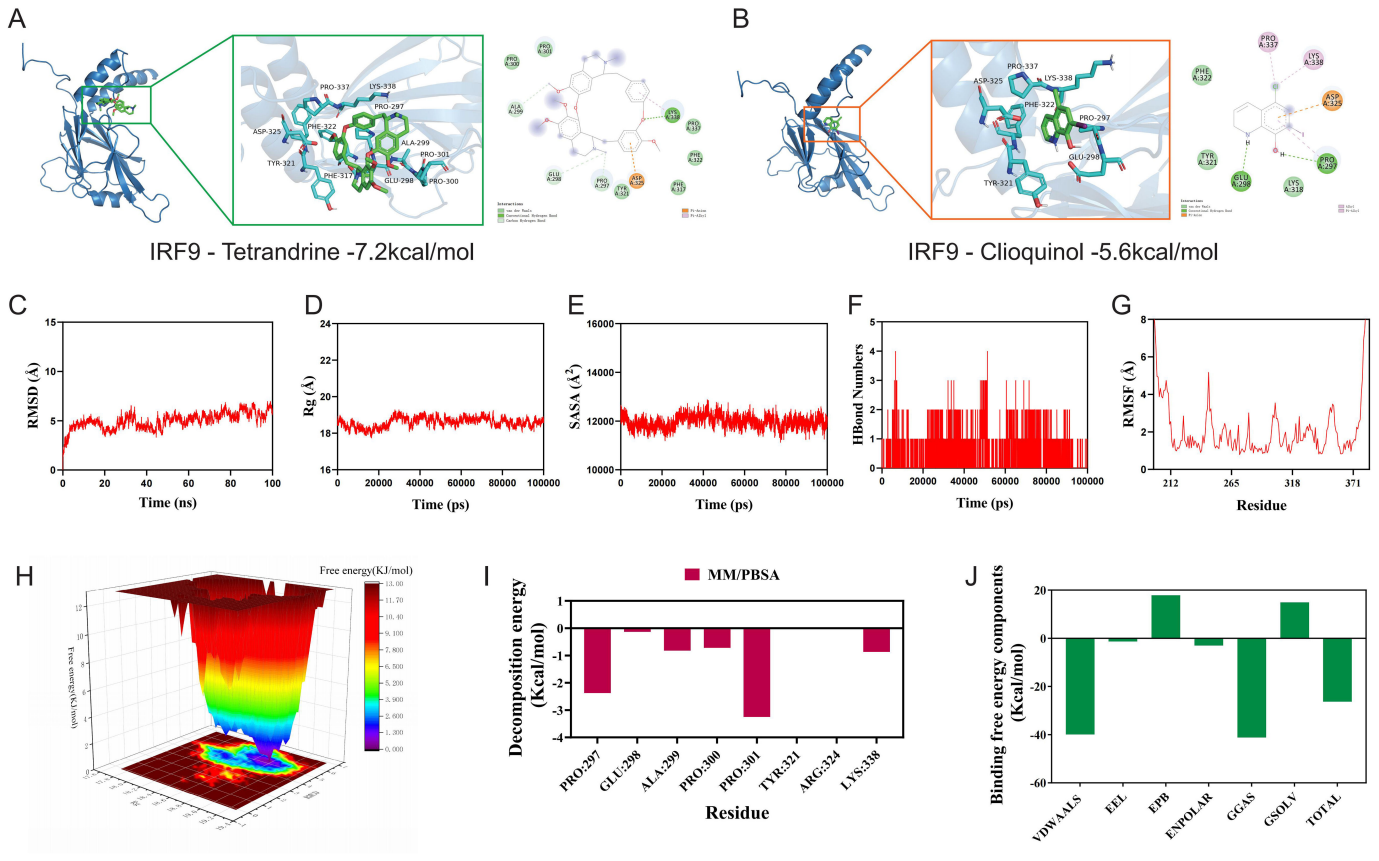
Molecular docking and MD simulation of IRF9 with candidate compounds. **(A)** Binding pose and interaction diagram of the IRF9-Tetrandrine complex; **(B)** Binding pose and interaction diagram of the IRF9-Clioquinol complex; **(C)** RMSD profiles of the complexes over time; **(D)** Time evolution of the protein radius of gyration (Rg); **(E)** Time evolution of the solvent-accessible surface area (SASA); **(F)** Time evolution of protein-ligand hydrogen bonds; **(G)** Residue-wise RMSF distribution; **(H)** Three-dimensional free energy landscape (FEL); **(I)** MM/PBSA per-residue binding free-energy decomposition; **(J)** MM/PBSA energy components and total binding free energy.

Given that IRF9 was identified as a potential therapeutic target in ccRCC, we next explored its small-molecule binding potential through molecular docking and dynamics simulations. Two candidate compounds—Tetrandrine and Clioquinol—were selected from preliminary virtual screening based on their predicted affinity for the IRF9 interface. Structural inspection of the docked complexes showed that both ligands occupied a pocket at the IRF9 interface and formed multiple hydrogen bonds and hydrophobic contacts (**Figures 6A, B**). RMSD analysis indicated that both complexes reached equilibrium and remained stable throughout the simulations (**Figure 6C**), while a nearly constant radius of gyration reflected compact protein conformations (**Figure 6D**). SASA varied only slightly, suggesting limited changes in solvent exposure upon ligand binding (**Figure 6E**). The number of protein–ligand hydrogen bonds was largely maintained during the trajectories, supporting persistent intermolecular contacts (**Figure 6F**). RMSF profiles revealed overall low residue flexibility, with fluctuations mainly in loop regions (**Figure 6G**). Free-energy landscape analysis further indicated a dominant low-energy basin, consistent with stable binding modes (**Figure 6H**). MM/PBSA per-residue decomposition identified key binding hotspots—PRO297, GLU298, ALA299, PRO300, PRO301, TYR321, ARG324, and LYS338 (**Figure 6I**). Component analysis showed that van der Waals and electrostatic interactions dominated favorable binding, whereas polar solvation opposed it, and the overall binding free energies estimated by MM/PBSA favored Tetrandrine over Clioquinol (ΔG_bind ≈ –7.2 vs. –5.6 kcal/mol) (**Figure 6J**), suggesting that Tetrandrine forms a more stable and energetically favorable interaction with IRF9. These findings highlight Tetrandrine as a potential small-molecule modulator of IRF9 activity, providing a structural basis for future drug design efforts targeting the PTM–immunity axis in ccRCC.

### Functional validation of IRF9 in promoting ccRCC progression

**Figure 7:**
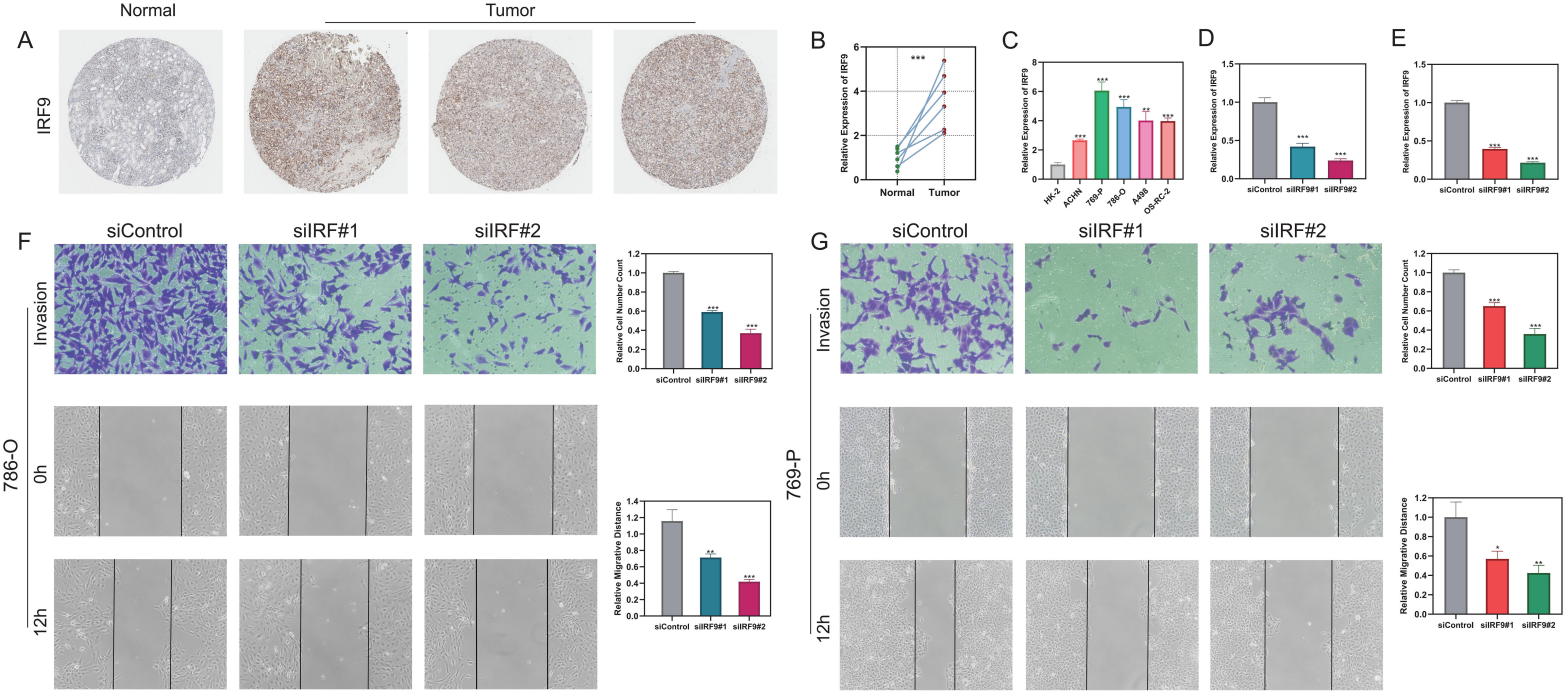
IRF9 expression and functional validation in ccRCC. **(A)** Immunohistochemical staining showing IRF9 expression in normal and tumor tissues. **(B)** Paired statistical analysis of IRF9 expression between tumor and adjacent normal tissues. **(C)** qPCR analysis of IRF9 expression across ccRCC cell lines. **(D, E)** Validation of IRF9 knockdown efficiency in 786-O and 769-P cells by siRNA. **(F)** Transwell invasion assay showing reduced invasive ability of 786-O cells after IRF9 knockdown. **(G)** Transwell and wound-healing assays indicating that IRF9 knockdown suppressed both invasion and migration in 769-P cells. *P<0.05, **P<0.01, ***P<0.001.

**Figure 8:**
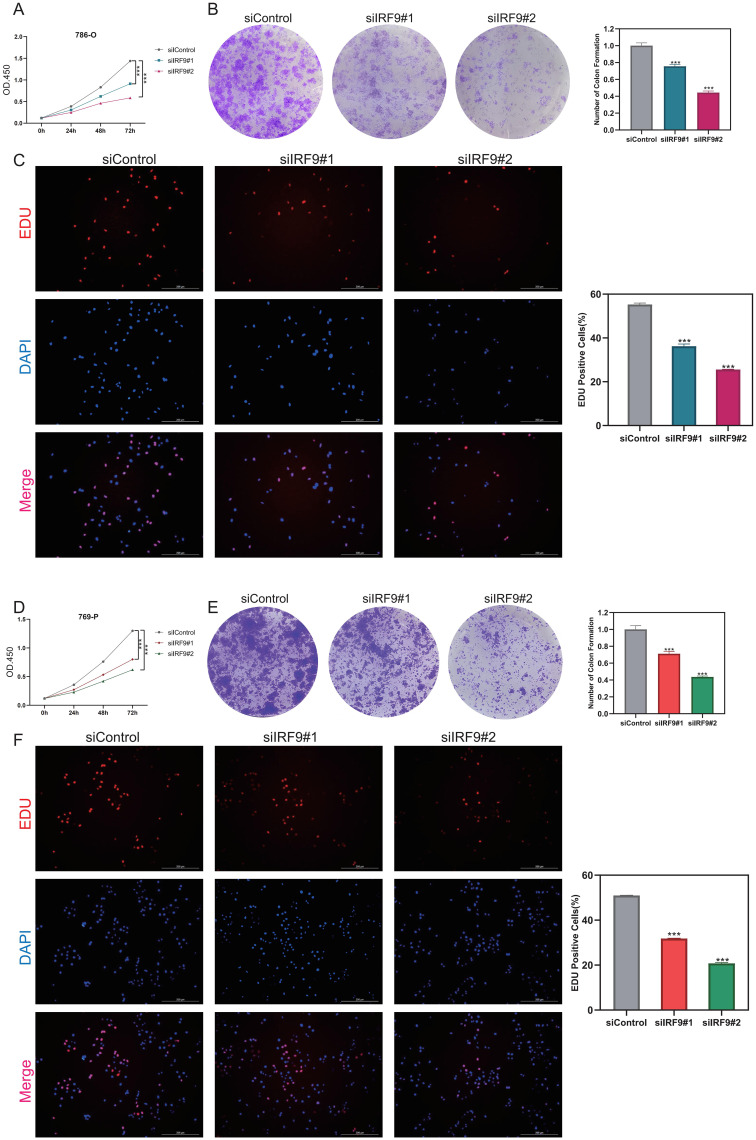
IRF9 knockdown suppressed proliferation of ccRCC cells. **(A, D)** CCK-8 assays showing reduced proliferation in 786-O and 769-P cells following IRF9 knockdown. **(B, E)** Colony formation assays indicating a significant decrease in colony numbers upon IRF9 silencing in 786-O and 769-P cells. **(C, F)** EdU assays demonstrating impaired DNA synthesis and proliferative activity after IRF9 knockdown in both cell lines. ***P<0.001.

We further validated the expression and functional contribution of IRF9 in ccRCC. Immunohistochemistry revealed markedly higher IRF9 levels in tumor than in normal tissues (**Figure 7A**), a finding corroborated by paired analyses of ccRCC specimens (**Figure 7B**). Consistently, IRF9 was highly expressed across multiple ccRCC cell lines (**Figure 7C**). Silencing IRF9 using siRNAs significantly reduced IRF9 expression in 786-O and 769-P cells (**Figures 7D** and **E**) and, as shown by transwell and wound-healing assays, significantly curtailed cellular invasion and migration (**Figures 7F, G**). To further evaluate the role of IRF9 in tumor cell growth, functional assays were performed. CCK-8 assays showed that silencing IRF9 significantly suppressed the proliferation rate of both 786-O (**Figure 8A**) and 769-P cells (**Figure 8D**). Consistently, colony formation assays revealed that IRF9 knockdown led to a marked decrease in colony numbers in 786-O (**Figure 8B**) and 769-P cells (**Figure 8E**). Moreover, EdU assays confirmed that suppression of IRF9 impaired DNA synthesis and proliferative capacity in 786-O (**Figure 8C**) and 769-P cells (**Figure 8F**). These findings collectively demonstrate that IRF9 functions as an oncogenic factor in ccRCC by enhancing tumor cell proliferation, invasion, and migration.

### IRF9 overexpression promotes proliferation and migration of ccRCC cells

**Figure 9:**
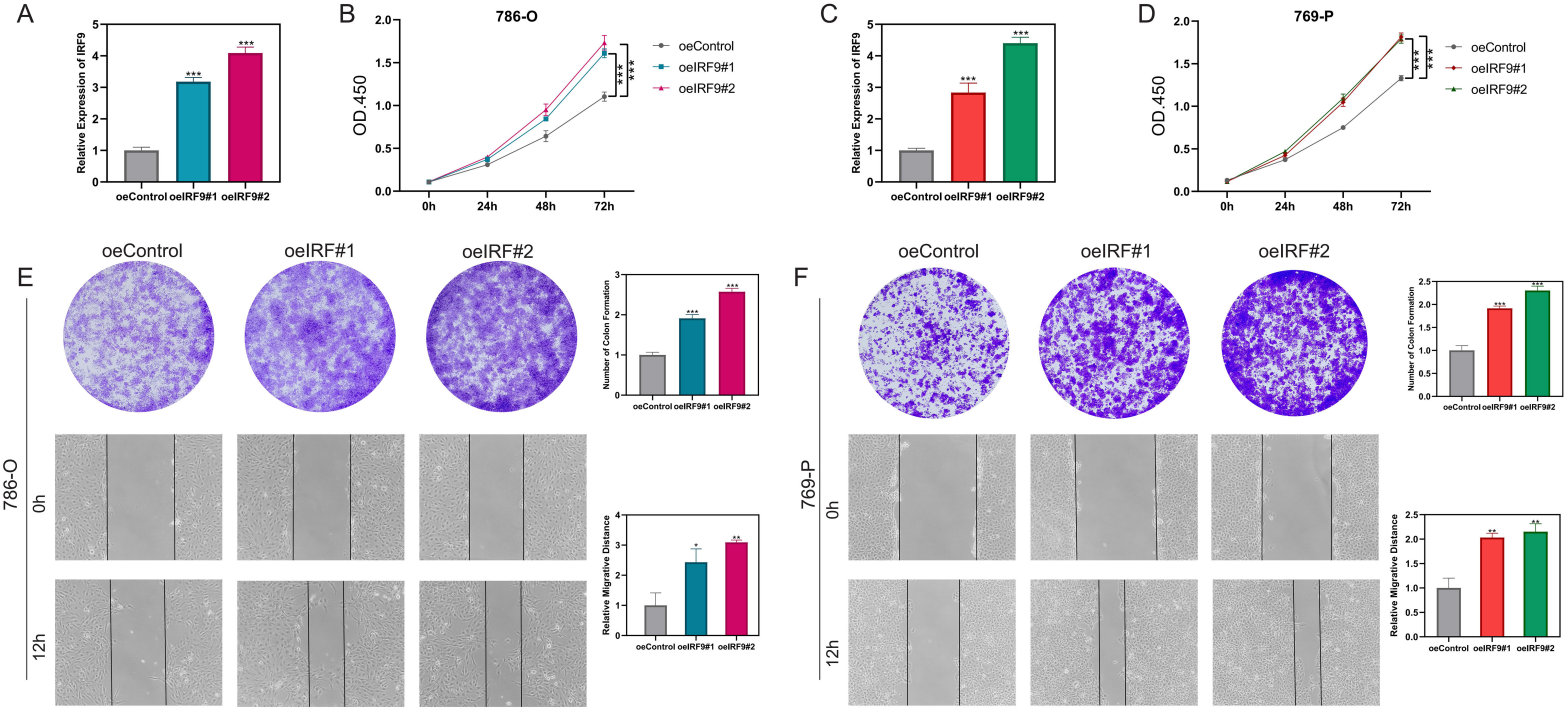
IRF9 overexpression promotes proliferation and migration in ccRCC cells. **(A, C)** qRT-PCR confirming successful IRF9 overexpression in 786-O and 769-P cells. **(B, D)** CCK-8 assays showing enhanced proliferation in IRF9-overexpressing cells. **(E, F)** Colony formation (upper panels) and wound-healing assays (lower panels) demonstrating increased growth and migratory ability after IRF9 overexpression. *P<.05, **P<0.01, ***P<0.001.

To substantiate IRF9’s functional impact, we conducted gain-of-function assays in ccRCC cell lines. Overexpression of IRF9 was successfully achieved in both 786-O and 769-P cells, as confirmed by qRT-PCR (**Figures 9A, C**). CCK-8 assays revealed that IRF9 overexpression significantly enhanced cell proliferation in both cell lines compared with controls (**Figures 9B, D**). Consistently, colony formation assays demonstrated an increased number of colonies upon IRF9 overexpression. Wound-healing assays further showed that enforced IRF9 expression markedly promoted the migratory capacity of 786-O and 769-P cells (**Figures 9E, F**). These findings provide strong evidence that IRF9 acts as a tumor-promoting factor in ccRCC by enhancing cell growth and motility.

## Discussion

In this study, we constructed and validated a novel PTMRS for ccRCC, integrating immune-related and PTM-related genes with transcriptomic data from TCGA-KIRC. Although the neural network model was not part of the final PTMRS, it served as a confirmatory analysis demonstrating that PTM-related transcriptomic features possess strong prognostic discriminatory ability, supporting their integration into the RSF-based PTMRS. Our model demonstrated strong predictive ability for patient prognosis and immunotherapy response. Single-cell transcriptomic analyses localized high PTMRS to immunosuppressive compartments, notably regulatory T cells and exhausted CD8^+^ T cells, consistent with the score reflecting functional states of the TME. Importantly, we identified IRF9 as a key PTMRS-associated gene, and functional experiments confirmed its role in promoting ccRCC proliferation, invasion, and migration. These findings provide novel insights into the interplay between PTMs and immune regulation in renal cancer.

PTMs are central regulators of protein activity, stability, and signaling, and their dysregulation has been linked to tumorigenesis and immune escape (19, 20). Recent evidence suggests that PTMs such as ubiquitination, phosphorylation, and acetylation modulate immune checkpoints and T-cell signaling pathways (21). In ccRCC, aberrant PTM regulation has been implicated in metabolic rewiring, immune suppression, and therapy resistance (17). By incorporating PTM-related genes into our model, we were able to capture these immunoregulatory layers, extending beyond conventional immune gene signatures. In the perioperative setting, PTMRS may assist in identifying patients at higher risk of postoperative recurrence who could benefit from intensified surveillance or adjuvant therapy. In the metastatic context, combining PTMRS with immune biomarkers such as PD-L1 or TMB could further refine patient stratification for immunotherapy responsiveness.

Our single-cell analysis revealed that PTMRS was significantly enriched in Tregs and exhausted CD8^+^T cells, both of which play essential roles in ccRCC progression. Tregs contribute to immune evasion by suppressing effector T-cell responses, while chronic antigen stimulation drives CD8^+^T cells toward an exhausted phenotype characterized by impaired cytotoxicity and upregulation of inhibitory receptors (22, 23). This is consistent with previous studies showing that ccRCC exhibits one of the most immunosuppressive tumor microenvironments among solid cancers (24, 25). In line with the concept that composite biology-based indices encode the immune landscape, Wei et al. derived a necroptosis index that stratified prognosis and delineated distinct TME infiltration patterns in ccRCC (26). Complementing our findings, Zhu et al. defined cholesterol-homeostasis–related ccRCC subtypes with divergent prognoses and immune characteristics and derived a validated cholesterol homeostasis signature, underscoring metabolic–immune crosstalk relevant to our PTMRS framework (27). In the perioperative setting, patients with high PTMRS/high TMB may merit closer surveillance and consideration of adjuvant approaches, as the immunosuppressive features captured by PTMRS could mitigate the expected benefit of high TMB. In the metastatic setting, high PTMRS/high TMB may favor combination regimens or clinical-trial enrollment, with PD-L1/TMB used alongside PTMRS to refine selection. Together, these observations support the idea that pathway-informed scores—PTMRS included—act as functional readouts of T-cell dysfunction and immunosuppression in ccRCC.

Among PTMRS genes, IRF9 emerged as a critical regulator. IRF9 forms the ISGF3 complex with STAT1/STAT2 and mediates type I interferon (IFN-I) signaling (28, 29). While IFN-I pathways can activate anti-tumor immunity, chronic IFN-I signaling has been shown to induce immune tolerance, promote PD-L1 expression, and drive resistance to immunotherapy (30, 31). Our results align with recent evidence that aberrant interferon signaling contributes to tumor aggressiveness and immune evasion (32). In functional assays, IRF9 knockdown impaired ccRCC proliferation and migration, whereas IRF9 overexpression enhanced these malignant traits, supporting its oncogenic role in renal cancer. Similar oncogenic properties of IRF9 have been reported in other tumor types, including lung cancer (33), colorectal cancer (34) and myeloid leukemia (35).

The clinical management of ccRCC has been transformed by ICIs, yet a substantial proportion of patients fail to respond (36, 37). Biomarkers such as PD-L1 expression and TMB are imperfect predictors of response (38, 39). Our findings suggest that PTMRS may serve as an additional biomarker, capturing the immunosuppressive state of the TME and predicting ICI responsiveness. Furthermore, IRF9 could represent a therapeutic target, as its modulation may enhance anti-tumor immunity and improve the efficacy of checkpoint blockade.

Our study has several limitations. First, it is based on retrospective transcriptomic datasets, and prospective validation in large clinical cohorts is required. Second, although we verified the function of IRF9 *in vitro*, *in vivo* studies are needed to confirm its role in immune regulation and therapy resistance. Third, the precise PTM mechanisms through which IRF9 contributes to ccRCC progression remain unclear and warrant further investigation. Integrating multi-omics approaches, such as phosphoproteomics and epigenomics, may provide deeper insights into PTM-dependent immune regulation. Finally, the predictive performance of PTMRS for immunotherapy response was inferred from retrospective transcriptomic data and Tumor Immune Dysfunction and Exclusion (TIDE) analysis rather than clinical outcomes. Prospective validation in well-characterized immunotherapy cohorts or clinical trials is needed to confirm its clinical relevance, and future integration with spatial or proteomic data may further elucidate its biological basis.

## Conclusion

In summary, we developed a PTM-related prognostic signature that predicts clinical outcome and immune status in ccRCC. PTMRS was enriched in immunosuppressive T-cell subsets, and IRF9 was identified as a tumor-promoting factor. These findings highlight the interplay between PTMs and tumor immunity, providing novel insights into prognostic stratification and therapeutic targeting in ccRCC.

## Data Availability

The original contributions presented in the study are included in the article/**Supplementary Material**. Further inquiries can be directed to the corresponding author.

## Supplementary material

Supplementary Figure 1 Neural network model and predictive performance. **(A)** Model architecture after 100 epochs with accuracy of 0.98 and loss of 0.05. **(B–D)** Training set performance with nearly perfect classification (AUC = 1.000). **(E–G)** Testing set performance: confusion matrix, prediction distribution, and ROC curve (AUC = 0.942). **(H–J)** External validation in GSE40435 showing good predictive ability (AUC = 0.906) with reduced specificity.
