# Supplementary material for: Integrative multi-omics analysis identifies a PTM-related immune signature and IRF9 as a driver in ccRCC
Source: Front Immunol. 2025 Dec 1;16:1707375. doi: 10.3389/fimmu.2025.1707375 (PMC12702869; doi:10.3389/fimmu.2025.1707375)
Supplement: Supplementary file 1 [file Table1.docx]

**Cell culture and siRNA transfection**

Human ccRCC cell lines (786-O and 769-P) and the normal renal tubular epithelial cell line HK-2 were obtained from ATCC. Cells were maintained in RPMI-1640 medium supplemented with 10% fetal bovine serum and 1% penicillin-streptomycin at 37℃ in a humidified atmosphere with 5% CO2. Small interfering RNAs (siRNAs) targeting IRF9 was transfected using Lipofectamine 3000 (Invitrogen) according to the manufacturer's protocol. Knockdown efficiency was confirmed by qRT-PCR. For overexpression experiments, cells were transfected with pcDNA3.1-based plasmids encoding full-length IRF9 (oeIRF9) or empty vector (oeControl) using Lipofectamine 3000. Stable overexpression efficiency was verified by qRT-PCR before subsequent functional assays.

**Cell proliferation assays**

Cell viability was evaluated using the Cell Counting Kit-8 (CCK-8, Beyotime, Shanghai, China) according to the manufacturer’s instructions. Colony formation assays were performed by seeding 1000 cells per well in six-well plates and culturing for 14 days before fixation and crystal violet staining. DNA synthesis capacity was assessed using an EdU incorporation assay kit (Beyotime, Shanghai, China) following the manufacturer’s protocol.

**Migration and invasion assays**

Cell migration was assessed using wound-healing assays. Briefly, cells were seeded in six-well plates, and a scratch was made using a sterile pipette tip; wound closure was monitored at 0 and 12h. Cell invasion was evaluated using Transwell chambers pre-coated with Matrigel. After 24 h of incubation, invasive cells were fixed, stained, and counted under a microscope.
