# Supplementary material for: Integrative multi-omics analysis identifies a PTM-related immune signature and IRF9 as a driver in ccRCC
Source: Front Immunol. 2025 Dec 1;16:1707375. doi: 10.3389/fimmu.2025.1707375 (PMC12702869; doi:10.3389/fimmu.2025.1707375)
Supplement: Supplementary file 2 [file Table2.docx]

| Immune Related Genes | PTM Related Genes | Dif Expressed Genes | Merge |
| --- | --- | --- | --- |
| AZGP1 | A2M | PRR35 | KNG1 |
| B2M | ABCC4 | SLC9A4 | ESRRB |
| CALR | ACKR3 | AQP2 | HRG |
| CANX | ACO1 | HS6ST2 | CHGB |
| CD1A | ACTG1 | MUC15 | GCGR |
| CD1B | ACVR1B | ACP3 | HSPA2 |
| CD1C | ACVRL1 | ATP12A | ANGPTL4 |
| CD1D | ADA2 | GPC5 | VIM |
| CD1E | ADAR | PIK3C2G | TYMP |
| CD4 | ADIPOQ | DUSP9 | TYROBP |
| CD8A | ADIPOR2 | TMEM207 | HLA-F |
| CD8B | ADRM1 | KNG1 | HLA-B |
| CD74 | AGT | TFAP2B | PSMB8 |
| CREB1 | AHNAK | KCNJ10 | FCGR3A |
| CTSB | AIMP1 | GP2 | HLA-A |
| CTSE | AKT1 | SLC12A1 | FCER1G |
| CTSL | AKT2 | ESRRB | C3 |
| CTSS | ANGPTL2 | UMOD | GMFG |
| FCER1G | ANGPTL3 | RALYL | CD40 |
| FCGRT | ANGPTL4 | HRG | TAPBP |
| PDIA3 | ANGPTL6 | TRPV6 | ISG20 |
| HFE | ANXA6 | FXYD4 | LCP2 |
| HLA-A | AP3B1 | IRX2 | PRF1 |
| HLA-B | APLNR | ELF5 | MYDGF |
| HLA-C | APOBEC3C | CA10 | ITGB2 |
| HLA-DMA | APOBEC3F | NELL1 | HMOX1 |
| HLA-DMB | APOD | CASR | BID |
| HLA-DOA | APOH | NRK | HCK |
| HLA-DOB | APOM | KCNJ1 | LILRB3 |
| HLA-DPA1 | AQP9 | TYRP1 | BTK |
| HLA-DPB1 | AR | CLDN16 | VAV1 |
| HLA-DQA1 | ARAF | OVCH2 | ITGAL |
| HLA-DQA2 | ARRB1 | ACOT12 | PML |
| HLA-DQB1 | AZGP1 | RANBP3L | BMP1 |
| HLA-DRA | AZU1 | IRX1 | GBP2 |
| HLA-DRB1 | B2M | MFSD4A | PDK1 |
| HLA-DRB3 | BCL10 | FAM3B | IL32 |
| HLA-DRB4 | BCL3 | COL4A6 | RAC2 |
| HLA-DRB5 | BECN1 | PTGER1 | PLXNB1 |
| HLA-E | BID | KCTD8 | HLA-DPB1 |
| HLA-F | BLNK | ERVMER34-1 | TAP1 |
| HLA-G | BMP1 | CLDN8 | CD3E |
| HLA-H | BPHL | SIM2 | ZAP70 |
| MR1 | BPI | CLUL1 | APOBEC3C |
| HSPA1A | BRAF | TPPP2 | SH3BP2 |
| HSPA1B | BRD8 | GGT6 | IL16 |
| HSPA1L | BST2 | RASL11B | INPP5D |
| HSPA2 | BTK | CLDN19 | CD4 |
| HSPA4 | C3 | NAT8L | HSPA6 |
| HSPA5 | C3AR1 | C4orf54 | PDGFRA |
| HSPA6 | C5 | SCNN1G | B2M |
| HSPA8 | C8G | GABRA2 | CD72 |
| HSP90AA1 | CACYBP | EHF | CD48 |
| HSP90AB1 | CALCRL | PLPPR1 | OGFR |
| ICAM1 | CALR | ADH1C | HLA-C |
| IFNA1 | CAMP | SPAG4 | HLA-DQB1 |
| IFNA2 | CANX | ADGRF3 | APOBEC3F |
| IFNA4 | CARD11 | MCCD1 | OAS1 |
| IFNA5 | CASP3 | BSND | ACKR3 |
| IFNA6 | CAT | NHLRC4 | PROCR |
| IFNA7 | CBL | RBP2 | CD74 |
| IFNA8 | CCR1 | HELT | CTSS |
| IFNA10 | CD14 | CALB1 | TGFB1 |
| IFNA13 | CD28 | CLCNKA | NR1H3 |
| IFNA14 | CD3E | TNNI1 | HLA-DMA |
| IFNA16 | CD4 | SFRP1 | FABP5 |
| IFNA17 | CD40 | UNCX | SORT1 |
| IFNA21 | CD48 | EGF | LTBR |
| IFNG | CD72 | SLC2A12 | C3AR1 |
| KIR2DL1 | CD74 | ADGRF1 | TNFRSF1A |
| KIR2DL2 | CD81 | EPN3 | HLA-DQA1 |
| KIR2DL3 | CDC42 | TMPRSS2 | HLA-G |
| KIR2DL4 | CDH1 | EDDM3A | MSR1 |
| KIR2DS1 | CDK4 | SERPINA5 | HLA-E |
| KIR2DS3 | CDNF | FLRT1 | PLXND1 |
| KIR2DS4 | CHGB | PAK6 | PTPRC |
| KIR2DS5 | CHP1 | PRDM16 | BLNK |
| KIR3DL1 | CHUK | NOS1AP | CD14 |
| KIR3DL2 | CLEC4M | EPB41L4B | HLA-DRB1 |
| KLRC1 | CMA1 | CA9 | TLR2 |
| KLRC2 | CMKLR1 | PRR15 | SH2D1A |
| KLRC3 | CMTM7 | MAPK4 | HLA-DPA1 |
| KLRD1 | CMTM8 | ERBB4 | HLA-DRA |
| LTA | CREB1 | HYKK | ISG15 |
| CIITA | CRLF3 | SCNN1B | CSF1R |
| MICA | CRP | BRINP3 | FAS |
| MICB | CSF1R | TMEM213 | RNASE2 |
| NFYA | CSK | FRMD7 | C5 |
| NFYB | CSRP1 | ARMH4 | BPHL |
| NFYC | CTSB | KLRG2 | PROC |
| LGMN | CTSG | NOS1 | CAT |
| PSMB8 | CTSL | CLCNKB | ANGPTL3 |
| PSMC1 | CTSS | TNNC1 | IL2RG |
| PSMC2 | CYBB | SLC4A9 | TRIM22 |
| PSMC3 | CYLD | SMIM5 | OSMR |
| PSMC4 | CYSLTR1 | GRHL2 | FCGR2B |
| PSMC5 | DAXX | SPTBN2 | PLCG2 |
| PSMC6 | DCK | F11 | CARD11 |
| PSMD1 | DDX17 | SLC4A1 | PRKCA |
| PSMD2 | DEFA3 | TMEM52B | S100A10 |
| PSMD3 | DEFA4 | FAM83B | TAP2 |
| PSMD4 | DES | WNK4 | CDH1 |
| PSMD5 | DUOX2 | FGF9 | PLXNC1 |
| PSMD7 | ECPAS | CCDC181 | SHC1 |
| PSMD8 | EDNRB | SLC12A3 | LYZ |
| PSMD10 | EGFR | PCDH9 | STING1 |
| PSMD11 | EIF2AK2 | KLHL14 | TLR8 |
| PSMD13 | ELANE | NR0B2 | ICAM1 |
| PSME1 | ELAVL1 | COL26A1 | INSR |
| PSME2 | ENG | GAL3ST3 | MMP9 |
| RELB | ERAP1 | MFSD6L | APOD |
| RFX5 | ERAP2 | SOST | FLT1 |
| RFXAP | ESRRB | PLA2G4F | MET |
| SLC10A2 | FABP3 | TMPRSS4 | BST2 |
| TAP1 | FABP4 | USP44 | NFATC2 |
| TAP2 | FABP5 | ADGRV1 | TEK |
| TAPBP | FAM3C | FOLR3 | IRF9 |
| THBS1 | FAS | FAM169A | PTAFR |
| SEM1 | FCER1G | SLC26A7 | NDRG1 |
| KLRC4 | FCGR2B | MYO3B | CYBB |
| AP3B1 | FCGR3A | ITLN1 | LCN2 |
| RFXANK | FCGRT | LRRC2 | FGFR2 |
| PSMD6 | FCN2 | SLC13A2 | NGFR |
| PSME3 | FGA | TMEM45B | HLA-DMB |
| PSMD14 | FGF2 | NDUFA4L2 | S100A8 |
| CLEC4M | FGFR1 | FAM167A | IL6R |
| IFI30 | FGFR2 | CHL1 | EGFR |
| PROCR | FGFR3 | ATP6V0A4 | NRP1 |
| ADRM1 | FLT1 | CHGB | TLR3 |
| ECPAS | FLT4 | TREM2 | MASP1 |
| TRPC4AP | FURIN | SLC9A2 | ANGPTL2 |
| CD209 | GBP2 | VTCN1 | VCAM1 |
| UBXN1 | GCGR | MRAP2 | APOH |
| ERAP1 | GDF15 | OXGR1 | APLNR |
| TAPBPL | GHR | SLC15A2 | SDC1 |
| KIR2DL5A | GMFB | OVOL2 | GDF15 |
| ERAP2 | GMFG | PROM2 | TNFRSF11B |
| ULBP3 | GNAI1 | ENTPD3 | OGN |
| ULBP2 | GPI | PRRG2 | HLA-DQA2 |
| ULBP1 | GRAP2 | ALDH3B2 | CCR1 |
| KIR3DL3 | GRB2 | VGLL1 | AQP9 |
| RAET1E | GREM2 | AQP6 | FLT4 |
| RAET1L | GRK2 | SEMG2 | AGT |
| UBR1 | GRN | ENPP6 | S100A9 |
| RAET1G | GSK3B | CEL | LTF |
| PDIA2 | HCK | TCF21 | MARCO |
| HAMP | HDAC1 | SYT7 | DES |
| PI3 | HDGF | RHCG | MAPT |
| CAMP | HDGFL3 | LHX1 | RBP4 |
| DEFB4A | HFE | KSR2 | AZGP1 |
| PPBP | HGF | SLC52A3 | ERAP2 |
| REG3G | HJV | FAM81A | SAA1 |
| CXCL14 | HLA-A | LMO3 |  |
| CXCL16 | HLA-B | DDN |  |
| SLPI | HLA-C | NOL3 |  |
| CXCL8 | HLA-DMA | GCGR |  |
| CXCL10 | HLA-DMB | GADL1 |  |
| CXCL9 | HLA-DPA1 | AC007906.2 |  |
| CXCL5 | HLA-DPB1 | BMP7 |  |
| CXCL11 | HLA-DQA1 | GPC3 |  |
| CXCL6 | HLA-DQA2 | PAPPA |  |
| CXCL1 | HLA-DQB1 | ATP6V1G3 |  |
| CXCL12 | HLA-DRA | KLK6 |  |
| CXCL13 | HLA-DRB1 | HSPA2 |  |
| CXCL2 | HLA-DRB3 | TAGLN3 |  |
| PF4 | HLA-DRB4 | CCNI2 |  |
| XCL1 | HLA-E | DMRT2 |  |
| CXCL3 | HLA-F | SLC4A11 |  |
| DEFB103B | HLA-G | CCSER1 |  |
| CCL13 | HLA-H | WNT9B |  |
| CCL1 | HMGB1 | GRM1 |  |
| DEFB1 | HMOX1 | NPHS2 |  |
| CCL8 | HNF4A | C1orf226 |  |
| ELANE | HRG | TMEM178A |  |
| DEFB103A | HSP90AA1 | FAM222A |  |
| DEFA3 | HSP90AB1 | FREM1 |  |
| DEFA1 | HSPA1B | CNTN1 |  |
| TMSB10 | HSPA2 | MPP7 |  |
| DEFA6 | HSPA4 | PROX1 |  |
| DEFA5 | HSPA5 | NUPR2 |  |
| DEFA4 | HSPA6 | RAB25 |  |
| LCN2 | HSPA8 | SUSD4 |  |
| LCN1 | ICAM1 | PPP1R1B |  |
| COLEC10 | ICAM2 | HILPDA |  |
| BPI | IFI30 | FOXI1 |  |
| S100A9 | IFIH1 | GATA3 |  |
| S100A8 | IFITM1 | TFCP2L1 |  |
| DCD | IFNAR1 | SIAH3 |  |
| LCN6 | IFNGR1 | HEPACAM2 |  |
| S100A12 | IGF2 | STC2 |  |
| HTN3 | IGF2R | SCN2A |  |
| LCN8 | IGHA1 | INPP5J |  |
| DEFA1B | IGHA2 | CYP2B6 |  |
| CCR10 | IGHG2 | RNF150 |  |
| CELA1 | IGHG3 | KLK7 |  |
| DEFB106A | IGHG4 | ATP6V0D2 |  |
| PENK | IGHM | SCN7A |  |
| BPIFC | IGHV1-18 | SCARB1 |  |
| MMP12 | IGHV1-2 | WNT8B |  |
| BPIFB6 | IGHV1-3 | NIPAL1 |  |
| LEAP2 | IGHV1-46 | FER1L6 |  |
| SFTPD | IGHV1-69 | MTURN |  |
| LCN9 | IGHV1-8 | MYLK3 |  |
| BPIFB2 | IGHV3-23 | APOC1 |  |
| PTGDS | IGHV3-43 | ANGPTL4 |  |
| TMSB4X | IGHV3-7 | TJP3 |  |
| PGLYRP1 | IGHV3-72 | LYPD6B |  |
| ZC3HAV1 | IGHV3-73 | CXCR4 |  |
| TMSB15A | IGHV4-34 | PRSS22 |  |
| S100B | IGHV5-51 | LIPH |  |
| S100A13 | IGKC | ATP6V1C2 |  |
| S100A6 | IGKV1-16 | TMEM61 |  |
| DEFB119 | IGKV1-17 | CACNA2D2 |  |
| DEFB107A | IGKV1-27 | S100A2 |  |
| DEFB105A | IGKV1-33 | RBBP8NL |  |
| SERPIND1 | IGKV1-5 | GRIK5 |  |
| DEFB129 | IGKV1-6 | C9orf135 |  |
| DEFB127 | IGKV1D-39 | TDGF1 |  |
| S100P | IGKV2-24 | TSPAN8 |  |
| S100A7 | IGKV2D-29 | ESRP1 |  |
| DEFB104A | IGKV3-15 | AMPH |  |
| DEFB126 | IGKV3-20 | NEXMIF |  |
| DEFB106B | IGKV3-7 | TRPV5 |  |
| DEFB104B | IGKV3D-11 | ANKRD34B |  |
| DEFB107B | IGKV4-1 | PPP2R2B |  |
| PGLYRP3 | IGKV6D-21 | MYBPH |  |
| PGLYRP2 | IGLC2 | TMEM30B |  |
| S100A10 | IGLC3 | MAP3K15 |  |
| S100A2 | IGLC7 | DDB2 |  |
| DEFB125 | IGLV1-47 | TTPA |  |
| DEFB123 | IGLV1-51 | NPHS1 |  |
| DEFB105B | IGLV3-1 | CWH43 |  |
| DEFB132 | IGLV3-19 | SRGAP3 |  |
| BPIFB3 | IGLV3-25 | ODF3B |  |
| LCN12 | IGLV3-9 | EYA4 |  |
| PGLYRP4 | IGLV6-57 | RASSF10 |  |
| S100A11 | IGLV7-46 | RHBG |  |
| S100A5 | IGLV9-49 | CHP2 |  |
| S100A3 | IKBKB | GSTM3 |  |
| S100A1 | IKBKG | AKAP3 |  |
| DEFB128 | IL10RB | VIM |  |
| DEFB108B | IL16 | ZNF488 |  |
| HTN1 | IL18 | C10orf82 |  |
| LMBR1L | IL18R1 | ASB15 |  |
| S100A7A | IL1R1 | ESRRG |  |
| DEFB118 | IL1RAP | NTNG1 |  |
| COLEC12 | IL1RN | SOWAHA |  |
| TMSB4Y | IL2RG | L1CAM |  |
| DEFB131A | IL32 | TTC36 |  |
| DEFB134 | IL6R | COL23A1 |  |
| DEFB130A | IL6ST | RNF212B |  |
| DEFB124 | ILK | ARRB2 |  |
| DEFB121 | INHBC | RIMBP2 |  |
| DEFB116 | INHBE | SLC4A8 |  |
| DEFB115 | INPP5D | MOGAT2 |  |
| DEFB114 | INSL6 | SPTB |  |
| DEFB113 | INSR | AVPR2 |  |
| DEFB112 | IREB2 | SLC6A17 |  |
| DEFB110 | IRF3 | TYMP |  |
| TMSB15B | IRF9 | SLC16A3 |  |
| DEFB133 | ISG15 | MPPED2 |  |
| S100Z | ISG20 | TYROBP |  |
| MAVS | ISG20L2 | PAPPA2 |  |
| TMSB4XP8 | ITGAL | SSC4D |  |
| S100A14 | ITGAV | CA8 |  |
| LCN10 | ITGB2 | DUSP26 |  |
| S100A16 | JAK1 | REEP6 |  |
| DEFB136 | JAK2 | ABHD17C |  |
| DEFB135 | JUN | HLA-F |  |
| DEFB117 | KLKB1 | RPS6KA6 |  |
| ZC3HAV1L | KNG1 | NNMT |  |
| S100A7L2 | KRAS | HMX2 |  |
| MBL3P | LANCL1 | PLPP4 |  |
| DEFB4B | LBP | TDRD5 |  |
| BPIFB4 | LCN2 | BMPR1B |  |
| IFNAR1 | LCP2 | HLA-B |  |
| AZU1 | LEAP2 | FGF1 |  |
| DEFB131B | LEPR | PEG3 |  |
| DEFA1A3 | LGMN | PCP4 |  |
| LCN1P1 | LILRB3 | FABP6 |  |
| S100G | LIMS1 | C16orf89 |  |
| DEFA7P | LPA | SAP30 |  |
| DEFB130B | LRP1 | EGLN3 |  |
| DEFB108F | LRSAM1 | IYD |  |
| DEFB131C | LTBP1 | CRHBP |  |
| TCHHL1 | LTBP3 | COBLL1 |  |
| TINAGL1 | LTBP4 | FRG2C |  |
| IFNGR1 | LTBR | LYPD6 |  |
| SLC22A17 | LTF | EMX1 |  |
| WFIKKN1 | LYN | ODAM |  |
| WFDC2 | LYZ | KCNK13 |  |
| IL6 | MALT1 | HOXB9 |  |
| UMODL1 | MANF | PRRT1B |  |
| TGFB1 | MAP2K1 | PSMB8 |  |
| PF4V1 | MAP2K2 | LILRB1 |  |
| MMP9 | MAPK1 | MAP6 |  |
| ANOS1 | MAPK14 | FMN2 |  |
| TLR4 | MAPK3 | RAB11FIP4 |  |
| SPAG11B | MAPK8 | DOK3 |  |
| A2M | MAPT | CPAMD8 |  |
| NFKB1 | MARCO | UPP2 |  |
| APOBEC3G | MASP1 | HK2 |  |
| FABP6 | MASP2 | RIPPLY1 |  |
| NOD2 | MAVS | TMSB10 |  |
| MBL2 | MBL2 | FOXJ1 |  |
| SFTPA1 | MET | HSF4 |  |
| RBP1 | MIF | CASZ1 |  |
| TLR2 | MMP9 | SCD |  |
| SLC40A1 | MPO | FCGR3A |  |
| PLAU | MSR1 | CDCA2 |  |
| IL1B | MX1 | CLDN14 |  |
| PAEP | MX2 | LMX1B |  |
| HJV | MYDGF | RHBDF2 |  |
| MUC5AC | NAMPT | ITGAX |  |
| OBP2A | NCK1 | ABCA4 |  |
| PLTP | NCK2 | ATP6V1B1 |  |
| MX1 | NDRG1 | NKD1 |  |
| DDX58 | NEDD4 | ANKRD2 |  |
| IFNL1 | NENF | DNASE1 |  |
| IRF3 | NEO1 | CDKN2A |  |
| SFTPA2 | NFATC1 | ATP1A1 |  |
| LPA | NFATC2 | SCNN1A |  |
| LBP | NFKB1 | DIO1 |  |
| RBP4 | NFKBIB | DNER |  |
| NOX4 | NFYA | MECOM |  |
| LTF | NFYB | CACNA2D3 |  |
| IFNB1 | NGFR | POU3F4 |  |
| RBP5 | NLRX1 | SH3GL2 |  |
| FABP7 | NR1H3 | ARHGAP22 |  |
| FABP5 | NR2C2 | SLC22A8 |  |
| FABP3 | NR2E3 | NXPH2 |  |
| FABP2 | NR2F1 | RPRM |  |
| FABP4 | NR3C1 | NETO2 |  |
| R3HDML | NRAS | SLC47A2 |  |
| BPIFA3 | NRP1 | CALCA |  |
| BPIFB1 | NUDT6 | B4GALNT2 |  |
| OASL | OAS1 | CAPSL |  |
| CRABP2 | OGFR | SLC25A35 |  |
| CRABP1 | OGN | ADM |  |
| RBP7 | ORM1 | NCCRP1 |  |
| DUOX1 | ORM2 | EFHD1 |  |
| OBP2B | OSMR | TUBB2B |  |
| RBP2 | PAK1 | DPP6 |  |
| LCN15 | PAK2 | DIRAS1 |  |
| CETP | PAK4 | SLC16A5 |  |
| FABP12 | PDF | SLC7A8 |  |
| FABP9 | PDGFRA | HLA-A |  |
| BPIFA1 | PDGFRB | RASAL3 |  |
| LCNL1 | PDIA3 | APELA |  |
| C8G | PDK1 | INHBB |  |
| SPAG11A | PF4 | RALGPS1 |  |
| PI15 | PGLYRP2 | ESM1 |  |
| NOX1 | PGRMC2 | TCEAL2 |  |
| PMP2 | PIK3CB | ARL4D |  |
| APOD | PIK3R1 | SEMA6D |  |
| ORM2 | PLCG1 | B4GALNT3 |  |
| ORM1 | PLCG2 | LGALS9 |  |
| TNF | PLSCR1 | HPCAL4 |  |
| CTSG | PLTP | TRPV2 |  |
| PRTN3 | PLXNA1 | TNFAIP6 |  |
| MAPK1 | PLXNA2 | CNKSR1 |  |
| PML | PLXNB1 | ANGPTL1 |  |
| AEN | PLXNB2 | SUCLG1 |  |
| CYBB | PLXNC1 | ENO2 |  |
| BPIFA2 | PLXND1 | PLCL1 |  |
| ISG20 | PML | VEGFA |  |
| BCL3 | PPBP | INSRR |  |
| ISG20L2 | PPIA | GABRD |  |
| NOX5 | PPP3CA | NUDT10 |  |
| NOX3 | PPP3R1 | COL5A3 |  |
| DUOX2 | PPP4C | ZNF728 |  |
| TLR3 | PRDX1 | ACSF2 |  |
| TFRC | PRDX2 | SLC7A13 |  |
| IFIH1 | PRF1 | C1orf116 |  |
| LRP1 | PRKCA | FECH |  |
| TRIM5 | PRKCB | PDE1A |  |
| IDO1 | PROC | PROZ |  |
| GDF15 | PROCR | OLFM4 |  |
| NEDD4 | PROK2 | ERMP1 |  |
| ADIPOQ | PRTN3 | STAC3 |  |
| STAT3 | PSMB8 | SHMT2 |  |
| STAT1 | PSMC1 | NAALADL2 |  |
| IFNL2 | PSMC2 | CD300A |  |
| SOCS3 | PSMC3 | MAB21L4 |  |
| SEMG1 | PSMC4 | FCER1G |  |
| TNFSF10 | PSMC5 | MAN1C1 |  |
| CCL20 | PSMC6 | TACSTD2 |  |
| SOCS1 | PSMD1 | C3 |  |
| RNASEL | PSMD10 | SYNE4 |  |
| IRF1 | PSMD11 | CAV2 |  |
| IL15 | PSMD13 | XPNPEP2 |  |
| APOBEC3F | PSMD14 | IL2RB |  |
| PLAAT4 | PSMD2 | SIX4 |  |
| CHIT1 | PSMD3 | PRKAR2B |  |
| CD40 | PSMD4 | MYO1F |  |
| TLR7 | PSMD5 | RNF43 |  |
| PPIA | PSMD6 | LY86 |  |
| ZYX | PSMD7 | SLC25A33 |  |
| NLRX1 | PSMD8 | SOSTDC1 |  |
| PGC | PSME1 | SPI1 |  |
| VEGFA | PSME2 | RGS19 |  |
| IKBKE | PSME3 | SSTR5 |  |
| ISG15 | PTAFR | DCXR |  |
| DHX58 | PTK2 | COLGALT1 |  |
| TNFAIP3 | PTK2B | SHISA2 |  |
| TFR2 | PTPN11 | SIM1 |  |
| FCN2 | PTPN6 | NR1I3 |  |
| MUC4 | PTPRC | CD70 |  |
| F2R | RABEP1 | WSCD2 |  |
| ELN | RABEP2 | PARVG |  |
| IL27 | RAC1 | KLHL13 |  |
| MAPT | RAC2 | AP1M2 |  |
| LYZ | RAC3 | FAM78A |  |
| CCL5 | RAF1 | LAPTM5 |  |
| LEP | RBP1 | C6orf52 |  |
| CYLD | RBP4 | KCTD1 |  |
| KLKB1 | RBP5 | LST1 |  |
| CST4 | RBP7 | SLC5A2 |  |
| CSRP1 | RELA | SLC14A2 |  |
| MAPK14 | RETN | SLC16A7 |  |
| JUN | RHOA | ST8SIA4 |  |
| ITGAV | RNASE2 | GPR182 |  |
| IRF5 | RNASE3 | LAMA4 |  |
| CCR6 | RNASEL | LGI2 |  |
| IL12B | ROBO1 | SEMA5B |  |
| TLR8 | RXRA | ACOT11 |  |
| GNLY | S100A10 | PIK3R5 |  |
| CD81 | S100A11 | VWA2 |  |
| EIF2AK2 | S100A12 | GMFG |  |
| APOM | S100A13 | NPY5R |  |
| CACYBP | S100A16 | FYB2 |  |
| NOD1 | S100A6 | HSD11B2 |  |
| MAPK8 | S100A8 | C2orf15 |  |
| MAPK3 | S100A9 | STRA6 |  |
| BST2 | S100B | PCCB |  |
| BPHL | S100P | ERP27 |  |
| PLA2G2A | SAA1 | PSMB9 |  |
| GRN | SBDS | IGFBP3 |  |
| NEWENTRY | SDC1 | SLC1A4 |  |
| PDGFRA | SDC2 | RBM11 |  |
| GNAI1 | SDC4 | COL4A5 |  |
| WNT5A | SEMA4B | SLC36A2 |  |
| FURIN | SEMA4C | CDH3 |  |
| ADAR | SEMA4D | GMPR |  |
| TYK2 | SEMA4G | RGS1 |  |
| NOS2 | SEMA7A | TNFSF9 |  |
| TRAF3 | SEPTIN7 | LZTS3 |  |
| TPT1 | SERPINA3 | TNFRSF4 |  |
| TPM2 | SERPIND1 | TNS4 |  |
| NEO1 | SH2D1A | IGSF6 |  |
| AHNAK | SH3BP2 | SIGLEC10 |  |
| TLR1 | SHC1 | CD40 |  |
| TK2 | SLC29A3 | HADH |  |
| PRDX2 | SLC40A1 | CD1D |  |
| MX2 | SOD1 | TNFRSF14 |  |
| FGF2 | SORT1 | LAIR1 |  |
| FGA | SOS1 | CD300LF |  |
| TCF7L2 | SP1 | NKG7 |  |
| F2RL1 | SRC | PLEKHB1 |  |
| TKFC | STAB2 | FMO5 |  |
| MSR1 | STAT1 | TAPBP |  |
| NFKBIZ | STAT3 | CCNP |  |
| LMBR1 | STING1 | ISG20 |  |
| EPPIN | SYK | GAL3ST4 |  |
| SRC | TANK | ANXA9 |  |
| MPO | TAP1 | RASD2 |  |
| ELAVL1 | TAP2 | BHLHE41 |  |
| ROBO3 | TAPBP | ENPP3 |  |
| SP1 | TAPBPL | AGAP2 |  |
| SOD1 | TBK1 | ZNF503 |  |
| PDF | TCF7L2 | GALNT3 |  |
| DLL4 | TEK | HOMER1 |  |
| ECD | TFR2 | RPS2 |  |
| SLC11A1 | TFRC | CLSTN2 |  |
| DMBT1 | TGFB1 | GPSM3 |  |
| STING1 | TGFBR2 | FBXO2 |  |
| SKIV2L | THBS1 | SELPLG |  |
| SEMG2 | TINAGL1 | ADORA3 |  |
| DES | TK2 | DTX2 |  |
| DCK | TKFC | LAT2 |  |
| DAXX | TLR1 | LCP2 |  |
| TNFRSF10A | TLR2 | DHRS11 |  |
| TNFRSF10B | TLR3 | CD37 |  |
| EED | TLR8 | TNFAIP8L2 |  |
| CCL4 | TMPRSS6 | CORO1A |  |
| LIMS1 | TMSB4X | TMEM91 |  |
| LALBA | TNC | KCNJ12 |  |
| APOBEC3H | TNFRSF11B | AQP5 |  |
| TMPRSS6 | TNFRSF1A | FMNL1 |  |
| SPINK5 | TNFSF13 | IKBIP |  |
| MARCO | TOR2A | TRADD |  |
| BECN1 | TPT1 | SLC16A1 |  |
| TNFSF11 | TRIM22 | ADAP2 |  |
| KNG1 | TRIM5 | MLKL |  |
| CSK | TUBB3 | SLC15A4 |  |
| KLRK1 | TXLNA | STAP1 |  |
| KCNH2 | TYK2 | HSPB7 |  |
| JUND | TYMP | C1QB |  |
| JAK1 | TYROBP | CTH |  |
| CLDN4 | UBR1 | E2F1 |  |
| CCL28 | UBXN1 | RUNX3 |  |
| RNASE3 | UNC93B1 | OSCAR |  |
| RN7SL1 | UTS2 | LSAMP |  |
| IRF7 | VAV1 | DTX1 |  |
| IREB2 | VAV2 | MT1H |  |
| ILK | VCAM1 | SQOR |  |
| IL18 | VIM | SLC48A1 |  |
| IL17A | VTN | TBL1Y |  |
| LTB4R | ZAP70 | FOXA3 |  |
| APOBEC3A | ZC3HAV1 | CCL5 |  |
| MASP2 | ZC3HAV1L | LRRN2 |  |
| TRIM27 | ZYX | GAS2L3 |  |
| RELA |  | HS6ST1 |  |
| IL7R |  | EPCAM |  |
| IL1A |  | C1orf162 |  |
| PTX3 |  | FSTL4 |  |
| IFNAR2 |  | GNLY |  |
| IFN1@ |  | PDP2 |  |
| SYTL1 |  | GZMH |  |
| APOBEC3C |  | C1QC |  |
| DDX17 |  | SHBG |  |
| PTGS2 |  | LGALS1 |  |
| HTR1A |  | SELENBP1 |  |
| SEPTIN7 |  | THRB |  |
| CD40LG |  | PRF1 |  |
| CD14 |  | CTSV |  |
| MASP1 |  | CAPS |  |
| PROC |  | MARVELD2 |  |
| MAP2K2 |  | PDLIM1 |  |
| MAP2K1 |  | SLC30A2 |  |
| HRG |  | IDO1 |  |
| NDRG1 |  | YBX3 |  |
| IRF9 |  | CYP4F2 |  |
| TRIM22 |  | MAL |  |
| LANCL1 |  | LILRB2 |  |
| PPP4C |  | PAQR7 |  |
| HMOX1 |  | SASH3 |  |
| HMGB1 |  | AGRP |  |
| RNASE7 |  | ZNF395 |  |
| ABCC4 |  | C1QA |  |
| HGF |  | MYDGF |  |
| HDAC1 |  | KRBA1 |  |
| IFNLR1 |  | ITGB2 |  |
| PLSCR1 |  | SIGLEC8 |  |
| BACH2 |  | MYOCOS |  |
| TANK |  | SDS |  |
| PIK3CG |  | PFKFB2 |  |
| ARRB1 |  | TBXAS1 |  |
| RSAD2 |  | IGSF11 |  |
| STAB2 |  | HCLS1 |  |
| TBK1 |  | STAMBPL1 |  |
| PDYN |  | ARHGDIB |  |
| PDGFRB |  | TUBAL3 |  |
| PDCD1 |  | TNFRSF10B |  |
| PCSK2 |  | PRDM1 |  |
| PCSK1 |  | ARRDC2 |  |
| ARG2 |  | DBT |  |
| AQP9 |  | CCM2 |  |
| FASLG |  | PIP4K2C |  |
| APOH |  | AGR2 |  |
| BIRC5 |  | DEF6 |  |
| ANXA6 |  | WAS |  |
| IL22 |  | CD247 |  |
| VTN |  | C5orf38 |  |
| VIM |  | GZMA |  |
| VCAM1 |  | ANGPT2 |  |
| PRDX1 |  | PFKFB4 |  |
| GFAP |  | SLC26A4 |  |
| GBP2 |  | PLOD3 |  |
| ALB |  | HMOX1 |  |
| SLC29A3 |  | FERMT3 |  |
| OAS1 |  | SFXN2 |  |
| AGER |  | GAS1 |  |
| UNC93B1 |  | LSP1 |  |
| TNFSF4 |  | EFHD2 |  |
| NOS1 |  | FOXC1 |  |
| ACTG1 |  | BBC3 |  |
| ACTA1 |  | GLOD5 |  |
| ACO1 |  | PDHB |  |
| SERPINA3 |  | CAV1 |  |
| CXCR1 |  | TRPM2 |  |
| CCL15 |  | GPAT3 |  |
| CCL14 |  | CCDC160 |  |
| CCL16 |  | DPP9 |  |
| CCL19 |  | GRAMD1A |  |
| CCL18 |  | SAMD12 |  |
| CCL17 |  | ACAA1 |  |
| CCL26 |  | ALDH6A1 |  |
| CCL22 |  | CST7 |  |
| CCR3 |  | CLDN11 |  |
| CCL4L1 |  | LILRB4 |  |
| ACKR2 |  | HECW1 |  |
| CCR7 |  | CR2 |  |
| CCL27 |  | NPTX2 |  |
| CCR8 |  | KCNQ1 |  |
| ACKR4 |  | AIF1L |  |
| CCL2 |  | PVALB |  |
| CCL21 |  | EHD2 |  |
| CCL7 |  | BID |  |
| CCL3 |  | DOK7 |  |
| CCL11 |  | MSC |  |
| CCR5 |  | EDA2R |  |
| CCL23 |  | NXPH4 |  |
| CCL25 |  | APBB1IP |  |
| CCL3L3 |  | PCGF1 |  |
| CCL4L2 |  | ASF1B |  |
| CCL3L1 |  | AHNAK2 |  |
| CCR1 |  | APOO |  |
| CCL24 |  | DGKD |  |
| XCL2 |  | LPCAT1 |  |
| CXCR4 |  | MPC1 |  |
| CXCR6 |  | PPM1H |  |
| CCR4 |  | SERPINA4 |  |
| TAFA5 |  | SLC9A3 |  |
| TAFA3 |  | DPEP2 |  |
| TAFA4 |  | RFC2 |  |
| TAFA1 |  | SLC37A2 |  |
| TAFA2 |  | ACKR2 |  |
| CCL15-CCL14 |  | BIN2 |  |
| PTK2B |  | PPM1K |  |
| IL4 |  | MTNR1A |  |
| CDH1 |  | CAND2 |  |
| LTBP1 |  | NLRC5 |  |
| IL13 |  | HPD |  |
| IL10 |  | CLEC2B |  |
| IL2 |  | BAX |  |
| PPARG |  | BDH1 |  |
| FGR |  | ALB |  |
| MIF |  | DEGS2 |  |
| CRP |  | C1QTNF6 |  |
| JAK2 |  | ANO5 |  |
| PTK2 |  | KCNJ13 |  |
| PTGDR |  | TRPM6 |  |
| CD86 |  | THEMIS2 |  |
| HCK |  | OVOL1 |  |
| VDR |  | RAP1GAP |  |
| OLR1 |  | PLAG1 |  |
| GRK2 |  | VWA5B1 |  |
| TXK |  | WNT7B |  |
| RNASE2 |  | CYFIP2 |  |
| CD79A |  | HCK |  |
| CD79B |  | COL4A4 |  |
| LYN |  | LAD1 |  |
| SYK |  | MS4A6A |  |
| BTK |  | MS4A7 |  |
| BLNK |  | CGN |  |
| VAV3 |  | KCNH3 |  |
| VAV1 |  | CLIC5 |  |
| VAV2 |  | PLXDC1 |  |
| RAC1 |  | EMP3 |  |
| RAC2 |  | THSD4 |  |
| RAC3 |  | OLFML2B |  |
| PPP3CA |  | CNTN3 |  |
| PPP3CB |  | SCN1B |  |
| PPP3CC |  | CARD16 |  |
| CHP1 |  | CBLC |  |
| PPP3R1 |  | MAGI3 |  |
| PPP3R2 |  | EPB41L5 |  |
| CHP2 |  | DGKZ |  |
| NFAT5 |  | PPP1R18 |  |
| NFATC1 |  | STX4 |  |
| NFATC2 |  | HSPB8 |  |
| NFATC3 |  | GGTA1 |  |
| NFATC4 |  | ABAT |  |
| HRAS |  | LILRB3 |  |
| KRAS |  | SLC13A3 |  |
| NRAS |  | CPNE6 |  |
| FOS |  | AK3 |  |
| CARD11 |  | LDHA |  |
| BCL10 |  | CD52 |  |
| MALT1 |  | AMFR |  |
| CHUK |  | CD2 |  |
| IKBKB |  | BTK |  |
| IKBKG |  | APOBEC3G |  |
| NFKBIA |  | FKBP11 |  |
| NFKBIB |  | PLEKHO1 |  |
| NFKBIE |  | CCDC88B |  |
| CD19 |  | CSF3R |  |
| CR2 |  | VAV1 |  |
| PIK3R5 |  | IFFO1 |  |
| PIK3R1 |  | ITGAL |  |
| PIK3R2 |  | GPD1L |  |
| PIK3R3 |  | APEH |  |
| PIK3CA |  | SLFN13 |  |
| PIK3CB |  | TFAP2A |  |
| PIK3CD |  | ST6GAL1 |  |
| AKT3 |  | ARHGEF39 |  |
| AKT1 |  | NFAM1 |  |
| AKT2 |  | GABARAPL1 |  |
| GSK3B |  | ARHGAP25 |  |
| INPP5D |  | TMC8 |  |
| CD22 |  | HOXB6 |  |
| CD72 |  | FATE1 |  |
| PTPN6 |  | SYP |  |
| LILRB3 |  | ERICH4 |  |
| FCGR2B |  | TMEM164 |  |
| RASGRP3 |  | NOP16 |  |
| PLCG2 |  | DACH1 |  |
| PRKCB |  | EVL |  |
| IFITM1 |  | PALD1 |  |
| IGH |  | MYO1G |  |
| IGHA1 |  | PTP4A3 |  |
| IGHA2 |  | FXYD5 |  |
| IGHD |  | RPS19 |  |
| IGHD1-1 |  | DEPP1 |  |
| IGHD1-14 |  | MATK |  |
| IGHD1-20 |  | ADAMTS16 |  |
| IGHD1-26 |  | FABP1 |  |
| IGHD1-7 |  | RNASET2 |  |
| IGHD2-15 |  | RELT |  |
| IGHD2-2 |  | NR3C2 |  |
| IGHD2-21 |  | CYP27B1 |  |
| IGHD2-8 |  | CTSW |  |
| IGHD3-10 |  | AEN |  |
| IGHD3-16 |  | S100A14 |  |
| IGHD3-22 |  | GPM6B |  |
| IGHD3-3 |  | BTN3A2 |  |
| IGHD3-9 |  | LOXL2 |  |
| IGHD4-11 |  | CDT1 |  |
| IGHD4-17 |  | FAM171A1 |  |
| IGHD4-23 |  | NCF4 |  |
| IGHD4-4 |  | TMCC1 |  |
| IGHD5-12 |  | PHYHD1 |  |
| IGHD5-18 |  | EBI3 |  |
| IGHD5-24 |  | RNF149 |  |
| IGHD5-5 |  | MT1G |  |
| IGHD6-13 |  | TYMS |  |
| IGHD6-19 |  | PLK2 |  |
| IGHD6-25 |  | CSTA |  |
| IGHD6-6 |  | WIPF1 |  |
| IGHD7-27 |  | CMTM4 |  |
| IGHE |  | ARHGAP9 |  |
| IGHG1 |  | EZH2 |  |
| IGHG2 |  | ADH1B |  |
| IGHG3 |  | CASP1 |  |
| IGHG4 |  | PML |  |
| IGHJ1 |  | PRDX4 |  |
| IGHJ2 |  | TBC1D24 |  |
| IGHJ3 |  | CYP17A1 |  |
| IGHJ4 |  | COL9A2 |  |
| IGHJ5 |  | CGREF1 |  |
| IGHJ6 |  | JAK3 |  |
| IGHM |  | BMP1 |  |
| IGHV1-18 |  | GBP2 |  |
| IGHV1-2 |  | PRAM1 |  |
| IGHV1-24 |  | AFM |  |
| IGHV1-3 |  | CORO2B |  |
| IGHV1-45 |  | NECTIN4 |  |
| IGHV1-46 |  | TRIB3 |  |
| IGHV1-58 |  | VWF |  |
| IGHV1-69 |  | KDF1 |  |
| IGHV1-8 |  | ILDR1 |  |
| IGHV1-38-4 |  | NUDT4 |  |
| IGHV1-69-2 |  | CSPG4 |  |
| IGHV2-26 |  | HOXD8 |  |
| IGHV2-5 |  | ALX1 |  |
| IGHV2-70 |  | BATF |  |
| IGHV3-11 |  | CAVIN3 |  |
| IGHV3-13 |  | PTPRE |  |
| IGHV3-15 |  | LARS2 |  |
| IGHV3-16 |  | CYTH4 |  |
| IGHV3-20 |  | PPP1R36 |  |
| IGHV3-21 |  | GLDC |  |
| IGHV3-23 |  | FCGR1A |  |
| IGHV3-30 |  | EPHA1 |  |
| IGHV3-30-3 |  | MAP3K9 |  |
| IGHV3-30-5 |  | FA2H |  |
| IGHV3-33 |  | MTCP1 |  |
| IGHV3-35 |  | PDK1 |  |
| IGHV3-38 |  | BTC |  |
| IGHV3-43 |  | GZMB |  |
| IGHV3-48 |  | PLA2G7 |  |
| IGHV3-49 |  | SVOPL |  |
| IGHV3-53 |  | LTB4R |  |
| IGHV3-64 |  | OXNAD1 |  |
| IGHV3-66 |  | POU5F1 |  |
| IGHV3-7 |  | PDHA1 |  |
| IGHV3-72 |  | EFNA3 |  |
| IGHV3-73 |  | IL32 |  |
| IGHV3-74 |  | VAT1L |  |
| IGHV3-9 |  | MPP2 |  |
| IGHV3-38-3 |  | SLC6A3 |  |
| IGHV3-69-1 |  | MRGPRF |  |
| IGHV4-28 |  | FKBP10 |  |
| IGHV4-30-1 |  | SLC38A3 |  |
| IGHV4-30-2 |  | PACRG |  |
| IGHV4-30-4 |  | TPX2 |  |
| IGHV4-31 |  | CSNK1E |  |
| IGHV4-34 |  | RAB7B |  |
| IGHV4-39 |  | ELMO1 |  |
| IGHV4-4 |  | PYGL |  |
| IGHV4-59 |  | FDX1 |  |
| IGHV4-61 |  | C4orf47 |  |
| IGHV4-38-2 |  | ATP6V1H |  |
| IGHV5-51 |  | GMIP |  |
| IGHV5-10-1 |  | PNMT |  |
| IGHV6-1 |  | P2RX7 |  |
| IGHV7-4-1 |  | ARHGAP15 |  |
| IGHV7-81 |  | KCNMB2 |  |
| IGK |  | NDC80 |  |
| IGKC |  | CD86 |  |
| IGKDEL |  | RAB37 |  |
| IGKJ |  | SLC29A2 |  |
| IGKJ1 |  | WNK3 |  |
| IGKJ2 |  | CENPU |  |
| IGKJ3 |  | CHST11 |  |
| IGKJ4 |  | FBXL8 |  |
| IGKJ5 |  | MCAM |  |
| IGKV@ |  | CD3D |  |
| IGKV1-12 |  | UNC13D |  |
| IGKV1-13 |  | C7 |  |
| IGKV1-16 |  | SCN2B |  |
| IGKV1-17 |  | PTTG1 |  |
| IGKV1-27 |  | MISP3 |  |
| IGKV1-33 |  | TRIM2 |  |
| IGKV1-37 |  | PLA2R1 |  |
| IGKV1-39 |  | SIGLEC9 |  |
| IGKV1-5 |  | SLC20A2 |  |
| IGKV1-6 |  | RBIS |  |
| IGKV1-8 |  | IL12RB1 |  |
| IGKV1-9 |  | PRELID2 |  |
| IGKV1D-12 |  | EVI2B |  |
| IGKV1D-13 |  | RAC2 |  |
| IGKV1D-16 |  | NEK6 |  |
| IGKV1D-17 |  | PHYKPL |  |
| IGKV1D-33 |  | PCYT2 |  |
| IGKV1D-37 |  | CCNA2 |  |
| IGKV1D-39 |  | GJC1 |  |
| IGKV1D-42 |  | TMEM140 |  |
| IGKV1D-43 |  | PHKA2 |  |
| IGKV1D-8 |  | RASSF2 |  |
| IGKV2-24 |  | NCKAP1L |  |
| IGKV2-28 |  | ATRNL1 |  |
| IGKV2-30 |  | P2RY1 |  |
| IGKV2-40 |  | RASSF4 |  |
| IGKV2D-24 |  | C3orf85 |  |
| IGKV2D-28 |  | GRIK3 |  |
| IGKV2D-29 |  | ITGA5 |  |
| IGKV2D-30 |  | GZMM |  |
| IGKV2D-40 |  | IL4R |  |
| IGKV3-11 |  | PSORS1C1 |  |
| IGKV3-15 |  | APOBEC3H |  |
| IGKV3-20 |  | ENOX1 |  |
| IGKV3-7 |  | GINS2 |  |
| IGKV3D-11 |  | CNGA1 |  |
| IGKV3D-15 |  | THOC6 |  |
| IGKV3D-20 |  | CASP4 |  |
| IGKV3D-7 |  | PLXNB1 |  |
| IGKV4-1 |  | TBX21 |  |
| IGKV5-2 |  | ALDH1A2 |  |
| IGKV6-21 |  | KLHL6 |  |
| IGKV6D-21 |  | TAF1D |  |
| IGKV6D-41 |  | CADPS2 |  |
| IGL |  | SYT1 |  |
| IGLC1 |  | CLEC2D |  |
| IGLC2 |  | RHOG |  |
| IGLC3 |  | DLX5 |  |
| IGLC6 |  | IL10RA |  |
| IGLC7 |  | RACK1 |  |
| IGLJ |  | UQCRC1 |  |
| IGLJ1 |  | SLA2 |  |
| IGLJ2 |  | PGF |  |
| IGLJ3 |  | NAP1L2 |  |
| IGLJ4 |  | HOXB5 |  |
| IGLJ5 |  | CDH16 |  |
| IGLJ6 |  | SLC5A11 |  |
| IGLJ7 |  | SHISA3 |  |
| IGLV@ |  | EXOSC5 |  |
| IGLV1-36 |  | HOXB8 |  |
| IGLV1-40 |  | HLA-DPB1 |  |
| IGLV1-44 |  | ETV7 |  |
| IGLV1-47 |  | DUSP15 |  |
| IGLV1-50 |  | FCGR2A |  |
| IGLV1-51 |  | ACTRT3 |  |
| IGLV10-54 |  | UBE2C |  |
| IGLV11-55 |  | BTN3A1 |  |
| IGLV2-11 |  | ADK |  |
| IGLV2-14 |  | CAB39L |  |
| IGLV2-18 |  | HLX |  |
| IGLV2-23 |  | TTYH3 |  |
| IGLV2-33 |  | METTL26 |  |
| IGLV2-8 |  | CARMIL1 |  |
| IGLV3-1 |  | NAV2 |  |
| IGLV3-10 |  | SPARC |  |
| IGLV3-12 |  | TMC4 |  |
| IGLV3-16 |  | KRT7 |  |
| IGLV3-19 |  | BTG2 |  |
| IGLV3-21 |  | RIN3 |  |
| IGLV3-22 |  | CGNL1 |  |
| IGLV3-25 |  | TAP1 |  |
| IGLV3-27 |  | PIK3R6 |  |
| IGLV3-32 |  | KIAA1522 |  |
| IGLV3-9 |  | PFKP |  |
| IGLV4-3 |  | ASPHD1 |  |
| IGLV4-60 |  | ABCC3 |  |
| IGLV4-69 |  | DPEP1 |  |
| IGLV5-37 |  | TMEM72 |  |
| IGLV5-39 |  | CROT |  |
| IGLV5-45 |  | APLN |  |
| IGLV5-48 |  | FGFBP1 |  |
| IGLV5-52 |  | ADA |  |
| IGLV6-57 |  | CYP2J2 |  |
| IGLV7-43 |  | MYO3A |  |
| IGLV7-46 |  | SHROOM3 |  |
| IGLV8-61 |  | CTXN3 |  |
| IGLV9-49 |  | TNFSF13B |  |
| C3 |  | RASD1 |  |
| C5 |  | MAL2 |  |
| CCL3P1 |  | CD3E |  |
| CKLF |  | LURAP1 |  |
| CMA1 |  | RDH10 |  |
| CX3CL1 |  | ORAI3 |  |
| CXCL17 |  | CTXND1 |  |
| CCN1 |  | TENT5B |  |
| EDN1 |  | ZAP70 |  |
| EDN2 |  | SFTA2 |  |
| EDN3 |  | KLC3 |  |
| FGF10 |  | DIRAS3 |  |
| LECT2 |  | ADAMTS7 |  |
| PPBPP1 |  | SERPINH1 |  |
| PROK2 |  | DEPTOR |  |
| SAA1 |  | CCL4 |  |
| SAA2 |  | DOK2 |  |
| SBDS |  | RHEBL1 |  |
| SEMA3A |  | PADI2 |  |
| SEMA3B |  | TRMT1 |  |
| SEMA3C |  | SFXN3 |  |
| SEMA3D |  | CAPN15 |  |
| SEMA3E |  | PTPN13 |  |
| SEMA3F |  | ZDHHC2 |  |
| SEMA3G |  | GLRX5 |  |
| SEMA4A |  | OCLN |  |
| SEMA4B |  | TOX3 |  |
| SEMA4C |  | NUDT1 |  |
| SEMA4D |  | APOBEC3C |  |
| SEMA4F |  | CENPM |  |
| SEMA4G |  | HK3 |  |
| SEMA5A |  | JPH4 |  |
| SEMA5B |  | ATP5F1A |  |
| SEMA6A |  | CKM |  |
| SEMA6B |  | ESRP2 |  |
| SEMA6C |  | PPP1R1A |  |
| SEMA6D |  | TMEM116 |  |
| SEMA7A |  | ACADSB |  |
| SLIT1 |  | SFXN5 |  |
| SLIT2 |  | ANXA4 |  |
| TNC |  | GOT2 |  |
| TYMP |  | FAM111B |  |
| C5AR1 |  | RAB42 |  |
| CCR9 |  | CHAC1 |  |
| CCRL2 |  | OPHN1 |  |
| CMKLR1 |  | ZMYND15 |  |
| CX3CR1 |  | AOAH |  |
| CXCR3 |  | SH3BP2 |  |
| CXCR5 |  | IL16 |  |
| ACKR3 |  | PRR15L |  |
| CYSLTR1 |  | CD6 |  |
| CYSLTR2 |  | RAPGEF3 |  |
| ACKR1 |  | GNA15 |  |
| EDNRA |  | LDHD |  |
| EDNRB |  | LZTS1 |  |
| FPR1 |  | IFNGR2 |  |
| FPR2 |  | C3orf52 |  |
| GPR17 |  | LPAR5 |  |
| GPR32 |  | CCBE1 |  |
| GPR33 |  | HSD3B7 |  |
| PTGDR2 |  | DNASE1L3 |  |
| C5AR2 |  | STAT4 |  |
| CXCR2 |  | INPP5D |  |
| LTB4R2 |  | MILR1 |  |
| PLAUR |  | LONRF2 |  |
| PLXNA1 |  | RIOX2 |  |
| PLXNA2 |  | WASF3 |  |
| PLXNA3 |  | GMNC |  |
| PLXNA4 |  | CPEB3 |  |
| PLXNB1 |  | TIMP1 |  |
| PLXNB2 |  | CD7 |  |
| PLXNB3 |  | LHFPL2 |  |
| PLXNC1 |  | THSD7A |  |
| PLXND1 |  | PNCK |  |
| PTAFR |  | SH3GL3 |  |
| ROBO1 |  | PPP1R3G |  |
| ROBO2 |  | MMP11 |  |
| RXFP3 |  | PNMA2 |  |
| XCR1 |  | SEPTIN1 |  |
| ADM |  | DCAF11 |  |
| ADM2 |  | RNF166 |  |
| AGRP |  | EVI2A |  |
| AGT |  | NUAK2 |  |
| AMBN |  | MRO |  |
| AMELX |  | SLC25A25 |  |
| AMH |  | AJM1 |  |
| ANGPTL5 |  | PTH1R |  |
| ANGPTL7 |  | TMIGD3 |  |
| APLN |  | PLIN2 |  |
| AREG |  | PLXNA4 |  |
| MANF |  | ARL11 |  |
| CDNF |  | CXCL9 |  |
| ARTN |  | S1PR4 |  |
| AVP |  | HAPLN3 |  |
| BDNF |  | INO80E |  |
| BMP1 |  | UQCRFS1 |  |
| BMP10 |  | TBC1D10C |  |
| BMP15 |  | ABLIM3 |  |
| BMP2 |  | AREL1 |  |
| BMP3 |  | BCAM |  |
| BMP4 |  | ARHGAP30 |  |
| BMP5 |  | TAGAP |  |
| BMP6 |  | RELB |  |
| BMP7 |  | LIMD2 |  |
| BMP8A |  | SNX20 |  |
| BMP8B |  | SEC61G |  |
| BTC |  | PPM1E |  |
| MYDGF |  | LOX |  |
| CALCA |  | CD4 |  |
| CALCB |  | PAG1 |  |
| CAT |  | SLC14A1 |  |
| CCK |  | FUT11 |  |
| CD320 |  | SMIM22 |  |
| CD70 |  | PLEK |  |
| ADA2 |  | SMIM10L2B |  |
| CER1 |  | BSPRY |  |
| CGA |  | LRRC52 |  |
| CGB3 |  | ACSL4 |  |
| CGB1 |  | HSPA6 |  |
| CGB2 |  | PDGFRA |  |
| CGB5 |  | EIF4EBP1 |  |
| CGB7 |  | PIP5K1B |  |
| CGB8 |  | PLCB2 |  |
| CHGA |  | DOK1 |  |
| CHGB |  | ERO1A |  |
| CLCF1 |  | TSPAN33 |  |
| CLEC11A |  | RASSF8 |  |
| CMTM1 |  | SLC1A3 |  |
| CMTM2 |  | TOP2A |  |
| CMTM3 |  | CAPN12 |  |
| CMTM4 |  | MELK |  |
| CMTM5 |  | CCR5 |  |
| CMTM6 |  | SUCLG2 |  |
| CMTM7 |  | BIRC5 |  |
| CMTM8 |  | TRIM9 |  |
| CNTF |  | ITGAM |  |
| CORT |  | B2M |  |
| CRH |  | ANK2 |  |
| CSF1 |  | MYBL2 |  |
| CSF2 |  | CD72 |  |
| CSF3 |  | CD48 |  |
| CSH1 |  | TMEM86A |  |
| CSH2 |  | ADRB1 |  |
| CSHL1 |  | IFI16 |  |
| CSPG5 |  | PSTPIP1 |  |
| CTF1 |  | IKZF1 |  |
| CCN2 |  | CD96 |  |
| DKK1 |  | OGFR |  |
| EBI3 |  | FGD2 |  |
| EGF |  | UXS1 |  |
| EPGN |  | CHSY3 |  |
| EPO |  | TRNP1 |  |
| EREG |  | XK |  |
| ESM1 |  | SLC34A1 |  |
| FAM3B |  | NDUFA4 |  |
| FAM3C |  | ARHGAP45 |  |
| FAM3D |  | PTPRO |  |
| FGF1 |  | SLC27A3 |  |
| FGF11 |  | UHRF1 |  |
| FGF12 |  | CLDN10 |  |
| FGF13 |  | CD84 |  |
| FGF14 |  | NDNF |  |
| FGF16 |  | HACD3 |  |
| FGF17 |  | HTATIP2 |  |
| FGF18 |  | REEP4 |  |
| FGF19 |  | OLFML2A |  |
| FGF20 |  | PALM |  |
| FGF21 |  | AGXT |  |
| FGF22 |  | RGS10 |  |
| FGF23 |  | MAP4K1 |  |
| FGF3 |  | HIGD1A |  |
| FGF4 |  | N4BP2L1 |  |
| FGF5 |  | C21orf62 |  |
| FGF6 |  | OR51E1 |  |
| FGF7 |  | CCL11 |  |
| FGF8 |  | SCIN |  |
| FGF9 |  | CSRNP3 |  |
| VEGFD |  | SLC30A8 |  |
| FIGNL2 |  | SULT2B1 |  |
| FLT3LG |  | CD300C |  |
| FSHB |  | CDK18 |  |
| GAL |  | PYCARD |  |
| GALP |  | ECHS1 |  |
| GAST |  | EMILIN2 |  |
| GCG |  | GAPDH |  |
| GDF1 |  | SLAMF6 |  |
| GDF10 |  | POLR3B |  |
| GDF11 |  | P4HA1 |  |
| GDF2 |  | PPP1R3C |  |
| GDF3 |  | ARHGAP24 |  |
| GDF5 |  | RAP2B |  |
| GDF6 |  | GAB3 |  |
| GDF7 |  | FAM151A |  |
| GDF9 |  | USP53 |  |
| GDNF |  | KIFC1 |  |
| GH1 |  | CD244 |  |
| GH2 |  | ITGB6 |  |
| GHRH |  | IGFBP2 |  |
| GHRL |  | SIGLEC1 |  |
| GIP |  | CDC45 |  |
| GKN1 |  | MOB3A |  |
| GMFB |  | CLEC7A |  |
| GMFG |  | PAQR5 |  |
| GNRH1 |  | HLA-C |  |
| GNRH2 |  | DTL |  |
| GPHA2 |  | SIRPB2 |  |
| GPHB5 |  | SGK2 |  |
| GPI |  | PCED1B |  |
| GREM1 |  | SORD |  |
| GREM2 |  | AP5M1 |  |
| GRP |  | MICAL1 |  |
| GUCA2A |  | KLHL3 |  |
| HBEGF |  | NCF1 |  |
| HDGF |  | LMBR1L |  |
| HDGFL3 |  | GAL3ST1 |  |
| IAPP |  | MBOAT2 |  |
| IFNE |  | DOC2A |  |
| IFNK |  | HLA-DQB1 |  |
| IFNW1 |  | DDIT4 |  |
| IGF1 |  | ANXA2R |  |
| IGF2 |  | CDO1 |  |
| IL11 |  | DCN |  |
| IL12A |  | CENPH |  |
| IL16 |  | APOBR |  |
| IL17B |  | BTBD16 |  |
| IL17C |  | VASH1 |  |
| IL17D |  | CSF2RA |  |
| IL17F |  | CTSH |  |
| IL19 |  | COL4A3 |  |
| IL1F10 |  | APOBEC3F |  |
| IL36RN |  | CD8A |  |
| IL36A |  | FAM180A |  |
| IL37 |  | CDKN2C |  |
| IL36B |  | P3H1 |  |
| IL36G |  | PEBP1 |  |
| IL1RN |  | SPC24 |  |
| IL20 |  | S1PR5 |  |
| IL21 |  | ST6GALNAC2 |  |
| IL23A |  | DLL4 |  |
| IL24 |  | KLK1 |  |
| IL25 |  | WNK1 |  |
| IL26 |  | RAB3B |  |
| IFNL3 |  | GM2A |  |
| IL3 |  | DDX41 |  |
| IL31 |  | IL18BP |  |
| IL32 |  | OAS1 |  |
| IL33 |  | MYO9B |  |
| IL34 |  | PTPN4 |  |
| IL5 |  | FCRL6 |  |
| IL6ST |  | CMTM3 |  |
| IL7 |  | CNP |  |
| IL9 |  | CXCR3 |  |
| INHA |  | STOX1 |  |
| INHBA |  | RPL18 |  |
| INHBB |  | BAG1 |  |
| INHBC |  | GSTO2 |  |
| INHBE |  | MS4A14 |  |
| INS |  | LDLRAD3 |  |
| INS-IGF2 |  | CD276 |  |
| INSL3 |  | LDHB |  |
| INSL4 |  | USP46 |  |
| INSL5 |  | ADTRP |  |
| INSL6 |  | TMEM44 |  |
| JAG1 |  | HRH2 |  |
| JAG2 |  | SH2B2 |  |
| FGF7P6 |  | DEFB1 |  |
| FGF7P3 |  | ACKR3 |  |
| KITLG |  | BATF3 |  |
| KL |  | ZNF205 |  |
| LACRT |  | PPARGC1A |  |
| LEFTY1 |  | PROCR |  |
| LEFTY2 |  | TMEM74B |  |
| LHB |  | CXCL10 |  |
| LIF |  | RBCK1 |  |
| LRSAM1 |  | SEZ6L2 |  |
| LTB |  | ATP6V0E2 |  |
| LTBP2 |  | AURKB |  |
| LTBP3 |  | SCPEP1 |  |
| LTBP4 |  | CEP55 |  |
| MDK |  | IL4I1 |  |
| MIA |  | SLC27A4 |  |
| MLN |  | EHBP1L1 |  |
| MSTN |  | MTCL1 |  |
| NAMPT |  | SLAMF7 |  |
| NDP |  | ABTB2 |  |
| NENF |  | TCAF2 |  |
| NGF |  | PARD3B |  |
| NMB |  | TBC1D4 |  |
| NODAL |  | RASGRP4 |  |
| CCN3 |  | RALGDS |  |
| NPFF |  | IQGAP3 |  |
| NPPA |  | PLEKHG2 |  |
| NPPB |  | PILRA |  |
| NPPC |  | BICDL1 |  |
| NPY |  | HCAR1 |  |
| NRG1 |  | FPR3 |  |
| NRG2 |  | HPGD |  |
| NRG3 |  | MXD3 |  |
| NRG4 |  | SRL |  |
| NRTN |  | BCL2A1 |  |
| NTF3 |  | FABP7 |  |
| NTF4 |  | BIK |  |
| NTS |  | SH3GL1 |  |
| NUDT6 |  | NCF2 |  |
| OGN |  | IRF7 |  |
| OSGIN1 |  | CD33 |  |
| OSM |  | CD74 |  |
| OSTN |  | CXCR6 |  |
| OXT |  | MPP5 |  |
| ENDOU |  | INSYN1 |  |
| PDGFA |  | ANXA3 |  |
| PDGFB |  | TCIRG1 |  |
| PDGFC |  | PRMT6 |  |
| PDGFD |  | KIF4A |  |
| PDGFRL |  | MYORG |  |
| PGF |  | TPD52L1 |  |
| PMCH |  | CTSS |  |
| PNOC |  | TGFB1 |  |
| POMC |  | KIF13B |  |
| PPBPP2 |  | HPCAL1 |  |
| PPY |  | STRA8 |  |
| PRL |  | AQP3 |  |
| PRLH |  | CTNNAL1 |  |
| PROK1 |  | TRIP13 |  |
| PSPN |  | SLC22A15 |  |
| PTH |  | PXDC1 |  |
| PTH2 |  | DOCK2 |  |
| PTHLH |  | VWA7 |  |
| PTN |  | RALBP1 |  |
| PYY |  | MICALL2 |  |
| QRFP |  | CD53 |  |
| RABEP1 |  | CDC6 |  |
| RABEP2 |  | SLC43A3 |  |
| REG1A |  | CRABP1 |  |
| RETN |  | FOXM1 |  |
| RETNLB |  | JAML |  |
| RLN1 |  | MKI67 |  |
| RLN2 |  | DISC1 |  |
| RLN3 |  | RDH8 |  |
| SCG2 |  | GABRP |  |
| SCGB3A1 |  | ABCD1 |  |
| SCT |  | SPN |  |
| AIMP1 |  | SNX33 |  |
| SECTM1 |  | BCKDHB |  |
| SLURP1 |  | NR1H3 |  |
| SPP1 |  | PHLDA3 |  |
| SST |  | PYHIN1 |  |
| STC1 |  | RPL28 |  |
| STC2 |  | ARID5A |  |
| TAC1 |  | CD5 |  |
| TDGF1 |  | TACC3 |  |
| TDGF1P3 |  | AMBP |  |
| TG |  | HLA-DMA |  |
| TGFA |  | SH2D2A |  |
| TGFB2 |  | UCHL1 |  |
| TGFB3 |  | CDKN2B |  |
| THPO |  | SKA3 |  |
| TNFRSF11B |  | FABP5 |  |
| TNFSF12 |  | MTMR10 |  |
| TNFSF13 |  | CD99 |  |
| TNFSF13B |  | SORT1 |  |
| TNFSF14 |  | PLCXD3 |  |
| TNFSF15 |  | PLAT |  |
| TNFSF18 |  | KLRB1 |  |
| TNFSF8 |  | LRRC25 |  |
| TNFSF9 |  | PNP |  |
| TOR2A |  | SMPDL3A |  |
| TRH |  | UBE2L6 |  |
| TSHB |  | HES4 |  |
| TSLP |  | IRF2BPL |  |
| TXLNA |  | CES4A |  |
| UCN |  | INTS6L |  |
| UCN2 |  | OXCT1 |  |
| UCN3 |  | LTBR |  |
| UTS2 |  | IL11 |  |
| UTS2B |  | POU2F2 |  |
| VEGFB |  | LPXN |  |
| VEGFC |  | PEPD |  |
| VGF |  | C3AR1 |  |
| VIP |  | SGPP1 |  |
| ACVR1B |  | PLAAT3 |  |
| ACVR1C |  | PRSS35 |  |
| ACVR2A |  | GGACT |  |
| ACVR2B |  | FDXR |  |
| ACVRL1 |  | MAN1A1 |  |
| ADCYAP1R1 |  | AP3S1 |  |
| ADIPOR1 |  | GTSE1 |  |
| ADIPOR2 |  | FAM110A |  |
| ADRB1 |  | DAG1 |  |
| ADRB2 |  | MYZAP |  |
| AGTR1 |  | TNFRSF18 |  |
| AGTR2 |  | TSPAN6 |  |
| AMHR2 |  | KIF21B |  |
| ANGPT1 |  | CTSZ |  |
| ANGPT4 |  | SPC25 |  |
| ANGPTL1 |  | CYRIA |  |
| ANGPTL2 |  | FAM219A |  |
| ANGPTL3 |  | ERBB2 |  |
| ANGPTL4 |  | TNFRSF1A |  |
| ANGPTL6 |  | SLC15A3 |  |
| APLNR |  | GCNT4 |  |
| AR |  | STK33 |  |
| AVPR1A |  | FHOD1 |  |
| AVPR1B |  | MUC3A |  |
| AVPR2 |  | SREBF2 |  |
| BMPR1A |  | TNFRSF1B |  |
| BMPR1B |  | HMGN5 |  |
| BMPR2 |  | SYT10 |  |
| BRD8 |  | RASA3 |  |
| C3AR1 |  | MNDA |  |
| CALCR |  | BAZ1A |  |
| CALCRL |  | CHRDL1 |  |
| CNTFR |  | MOB1B |  |
| CRHR1 |  | BARX2 |  |
| CRHR2 |  | SIT1 |  |
| CRIM1 |  | ALDOB |  |
| CRLF1 |  | LRRC43 |  |
| CRLF2 |  | HOGA1 |  |
| CRLF3 |  | NT5DC3 |  |
| CSF1R |  | MZT2A |  |
| CSF2RA |  | LCP1 |  |
| CSF2RB |  | ZNF44 |  |
| CSF3R |  | NLRC4 |  |
| EGFR |  | SBK1 |  |
| ENG |  | TBX3 |  |
| EPOR |  | SYNJ2BP |  |
| ESR1 |  | KCNE3 |  |
| ESR2 |  | ALS2CL |  |
| ESRRA |  | RASSF5 |  |
| ESRRB |  | PRR7 |  |
| ESRRG |  | FJX1 |  |
| FGFR1 |  | UCN |  |
| FGFR2 |  | HPSE2 |  |
| FGFR3 |  | PLEKHN1 |  |
| FGFR4 |  | MYH14 |  |
| FGFRL1 |  | SPACA9 |  |
| FLT1 |  | PLD4 |  |
| FLT3 |  | SLC39A4 |  |
| FLT4 |  | FASLG |  |
| FSHR |  | BRCC3 |  |
| GALR2 |  | RPL36 |  |
| GALR3 |  | ACAP1 |  |
| GCGR |  | NRP2 |  |
| GHR |  | UGT8 |  |
| GHRHR |  | EBF2 |  |
| GHSR |  | STMN3 |  |
| GIPR |  | TGFBR3 |  |
| GLP1R |  | GPR65 |  |
| GLP2R |  | COL6A2 |  |
| GNRHR |  | SALL3 |  |
| GPER1 |  | TESPA1 |  |
| HNF4A |  | ARNT2 |  |
| HNF4G |  | KIF20A |  |
| HTR3A |  | S100A5 |  |
| HTR3B |  | GPR27 |  |
| HTR3C |  | LILRA6 |  |
| HTR3D |  | RPL10 |  |
| HTR3E |  | POMGNT2 |  |
| IFNGR2 |  | NAT2 |  |
| IGF1R |  | EVPL |  |
| IGF2R |  | FADS3 |  |
| IL10RA |  | RPS14 |  |
| IL10RB |  | ARG2 |  |
| IL11RA |  | PLIN5 |  |
| IL12RB1 |  | UGT3A2 |  |
| IL12RB2 |  | PXK |  |
| IL13RA1 |  | PARD6A |  |
| IL13RA2 |  | OGDHL |  |
| IL15RA |  | ABCG1 |  |
| IL2RB |  | PPP1R13L |  |
| IL17RA |  | ZNF683 |  |
| IL17RB |  | CAMKK1 |  |
| IL17RC |  | HLA-DQA1 |  |
| IL17RD |  | PPFIA4 |  |
| IL17RE |  | RHOH |  |
| IL18R1 |  | ATP6V1A |  |
| IL18RAP |  | FBXO17 |  |
| IL1R1 |  | PALM3 |  |
| IL1R2 |  | GBP5 |  |
| IL1RAP |  | GRAMD1C |  |
| IL1RL1 |  | CCDC186 |  |
| IL1RL2 |  | CYGB |  |
| IL20RA |  | TAC1 |  |
| IL20RB |  | NPNT |  |
| IL21R |  | MAMDC2 |  |
| IL22RA1 |  | MS4A4A |  |
| IL22RA2 |  | MTOR |  |
| IL23R |  | RPL18A |  |
| IL27RA |  | RCN1 |  |
| IL2RA |  | SEMA6A |  |
| IL2RG |  | FAM193B |  |
| IL31RA |  | RIN1 |  |
| IL3RA |  | CYP39A1 |  |
| IL4R |  | ALOX5 |  |
| IL5RA |  | MCM5 |  |
| IL6R |  | ANKRD9 |  |
| IL9R |  | MAP3K7CL |  |
| INSR |  | HAPLN1 |  |
| KDR |  | C1orf54 |  |
| LEPR |  | IL15RA |  |
| LGR4 |  | P4HB |  |
| LGR5 |  | PDIA5 |  |
| LGR6 |  | MYH10 |  |
| LHCGR |  | SIPA1 |  |
| LIFR |  | CADM4 |  |
| LTBR |  | TCIM |  |
| MC1R |  | SCGB2A1 |  |
| MC2R |  | KISS1R |  |
| MC3R |  | BCL2L10 |  |
| MC4R |  | GDF6 |  |
| MCHR1 |  | NINL |  |
| MCHR2 |  | AKNA |  |
| MET |  | GAPT |  |
| MLNR |  | HMGCS2 |  |
| MPL |  | RPL13 |  |
| MTNR1A |  | TSPYL5 |  |
| MTNR1B |  | GRB14 |  |
| NGFR |  | TMEM233 |  |
| NMBR |  | AFAP1L2 |  |
| NPR1 |  | ADAMTS15 |  |
| NPR3 |  | GPRIN1 |  |
| NR0B1 |  | RUNX1 |  |
| NR0B2 |  | CDKL1 |  |
| NR1D1 |  | CFAP221 |  |
| NR1D2 |  | RRM2 |  |
| NR1H2 |  | HLA-G |  |
| NR1H3 |  | RAPGEFL1 |  |
| NR1H4 |  | ADAMTS4 |  |
| NR1I2 |  | RNASE6 |  |
| NR1I3 |  | VSIG1 |  |
| NR2C1 |  | LRRK1 |  |
| NR2C2 |  | ATG16L2 |  |
| NR2E1 |  | SYTL5 |  |
| NR2E3 |  | FGR |  |
| NR2F1 |  | SLC38A4 |  |
| NR2F2 |  | CLEC12A |  |
| NR2F6 |  | PAQR4 |  |
| NR3C1 |  | HVCN1 |  |
| NR3C2 |  | MOXD1 |  |
| NR4A1 |  | SLC25A5 |  |
| NR4A2 |  | CLK4 |  |
| NR4A3 |  | NDUFS1 |  |
| NR5A1 |  | ENAM |  |
| NR5A2 |  | PTGER3 |  |
| NR6A1 |  | TLCD1 |  |
| NRP1 |  | TSPAN4 |  |
| NRP2 |  | ARHGEF38 |  |
| OGFR |  | C1QTNF7 |  |
| OPRD1 |  | TBX19 |  |
| OPRK1 |  | CYSTM1 |  |
| OPRL1 |  | MISP |  |
| OPRM1 |  | SLA |  |
| OSMR |  | COX4I2 |  |
| OXTR |  | SAMHD1 |  |
| PGR |  | SLC2A1 |  |
| PGRMC2 |  | GNG7 |  |
| PPARA |  | NFKBIE |  |
| PPARD |  | MCOLN3 |  |
| PRLHR |  | PRR11 |  |
| PRLR |  | SERPINB9 |  |
| PTGER1 |  | IL21R |  |
| PTGER2 |  | SVIP |  |
| PTGER3 |  | KIAA0895L |  |
| PTGER4 |  | HJURP |  |
| PTGFR |  | FCMR |  |
| PTH1R |  | SLC2A3 |  |
| PTH2R |  | LLGL2 |  |
| RARA |  | TNK2 |  |
| RARB |  | KCNE4 |  |
| RARG |  | HLA-DQB2 |  |
| RORA |  | CTDSPL |  |
| RORB |  | CD27 |  |
| RORC |  | MSR1 |  |
| RXFP1 |  | FCHO1 |  |
| RXFP2 |  | SLFN11 |  |
| RXRA |  | SCIMP |  |
| RXRB |  | JAG2 |  |
| RXRG |  | MCUB |  |
| S1PR1 |  | SLC16A10 |  |
| S1PR2 |  | STK10 |  |
| SCTR |  | DIRAS2 |  |
| SDC1 |  | DUSP4 |  |
| SDC2 |  | TNNI2 |  |
| SDC3 |  | SLC44A4 |  |
| SDC4 |  | BIRC3 |  |
| SORT1 |  | STAB1 |  |
| SSTR1 |  | SLC22A7 |  |
| SSTR2 |  | HLA-E |  |
| SSTR5 |  | TLCD3A |  |
| ST2 |  | ZNF581 |  |
| TACR1 |  | SLC5A3 |  |
| TEK |  | RPLP0 |  |
| TGFBR1 |  | PCSK6 |  |
| TGFBR2 |  | PCCA |  |
| TGFBR3 |  | DNAH11 |  |
| THRA |  | COL4A1 |  |
| THRB |  | ITGA4 |  |
| TIE1 |  | PLXND1 |  |
| TNFRSF10C |  | PTPN22 |  |
| TNFRSF10D |  | HOXA7 |  |
| TNFRSF11A |  | CCND1 |  |
| TNFRSF12A |  | TYRO3 |  |
| TNFRSF13B |  | BTBD19 |  |
| TNFRSF13C |  | ALAS1 |  |
| TNFRSF14 |  | ZMYND12 |  |
| TNFRSF17 |  | PREX1 |  |
| TNFRSF18 |  | MSI2 |  |
| TNFRSF19 |  | OAT |  |
| TNFRSF1A |  | AK7 |  |
| TNFRSF1B |  | FBP1 |  |
| TNFRSF21 |  | PSMB10 |  |
| TNFRSF25 |  | CDKN3 |  |
| TNFRSF4 |  | NRG3 |  |
| TNFRSF6B |  | PLAU |  |
| TNFRSF8 |  | ALAD |  |
| TNFRSF9 |  | PSAT1 |  |
| TRHR |  | LNX1 |  |
| TSHR |  | MFSD3 |  |
| TUBB3 |  | HPCA |  |
| VIPR1 |  | ASAP3 |  |
| VIPR2 |  | ABCA1 |  |
| PTPN11 |  | SUSD1 |  |
| ICAM2 |  | CCNB2 |  |
| ITGAL |  | CD200 |  |
| ITGB2 |  | VSIR |  |
| PAK1 |  | RPS8 |  |
| NCR2 |  | DPT |  |
| TYROBP |  | ITPR2 |  |
| LCK |  | PTPRC |  |
| FCGR3A |  | BLNK |  |
| FCGR3B |  | ARHGEF1 |  |
| NCR1 |  | NCR3 |  |
| NCR3 |  | CXCL11 |  |
| CD247 |  | PNMA8A |  |
| ZAP70 |  | MFAP3L |  |
| LCP2 |  | IL17RE |  |
| LAT |  | HUNK |  |
| PLCG1 |  | IKZF3 |  |
| SH3BP2 |  | PDE1B |  |
| FYN |  | XCL2 |  |
| SHC2 |  | IL3RA |  |
| SHC4 |  | SAMD14 |  |
| SHC3 |  | EMB |  |
| SHC1 |  | AXL |  |
| GRB2 |  | FRK |  |
| SOS1 |  | PLXNA3 |  |
| SOS2 |  | CD3G |  |
| ARAF |  | GPRC5A |  |
| BRAF |  | SCOC |  |
| RAF1 |  | CD14 |  |
| HCST |  | GATA2 |  |
| CD48 |  | ANXA1 |  |
| CD244 |  | RPL35 |  |
| PRKCA |  | GPR4 |  |
| PRKCG |  | RGS14 |  |
| SH2D1B |  | TMEM45A |  |
| SH2D1A |  | ZHX3 |  |
| FAS |  | C16orf54 |  |
| GZMB |  | LILRA2 |  |
| PRF1 |  | JMY |  |
| CASP3 |  | CD180 |  |
| BID |  | SLC29A4 |  |
| CD3D |  | MARVELD3 |  |
| CD3E |  | NTRK2 |  |
| CD3G |  | RAB33A |  |
| PTPRC |  | RAB24 |  |
| ITK |  | LRRC19 |  |
| TEC |  | RPL22L1 |  |
| NCK1 |  | CRB2 |  |
| NCK2 |  | EOMES |  |
| GRAP2 |  | HLA-DRB1 |  |
| PAK2 |  | GRB10 |  |
| PAK3 |  | GNRH1 |  |
| PAK4 |  | BUB1 |  |
| PAK6 |  | GDF7 |  |
| PAK5 |  | PM20D1 |  |
| RHOA |  | TNFSF8 |  |
| CDC42 |  | PYGO1 |  |
| CD28 |  | NNT |  |
| ICOS |  | SLC11A1 |  |
| MAP3K8 |  | CEMIP2 |  |
| MAP3K14 |  | ENTPD1 |  |
| CTLA4 |  | TNFAIP3 |  |
| CBLC |  | SIRPG |  |
| CBL |  | PTGFR |  |
| CBLB |  | TROAP |  |
| CDK4 |  | MICB |  |
| RASGRP1 |  | DLGAP5 |  |
| PDK1 |  | TPI1 |  |
| PRKCQ |  | MYC |  |
| TRAC |  | CRTAM |  |
| TRAJ1 |  | ADAM8 |  |
| TRAJ2 |  | TRPA1 |  |
| TRAJ3 |  | ZNF692 |  |
| TRAJ4 |  | PBK |  |
| TRAJ5 |  | TLR2 |  |
| TRAJ6 |  | CGAS |  |
| TRAJ7 |  | SCD5 |  |
| TRAJ8 |  | SH2D1A |  |
| TRAJ9 |  | IRF6 |  |
| TRAJ10 |  | SLC17A9 |  |
| TRAJ11 |  | CLNK |  |
| TRAJ12 |  | SLC35G2 |  |
| TRAJ13 |  | MAP4K4 |  |
| TRAJ14 |  | TENM1 |  |
| TRAJ15 |  | ATP1B1 |  |
| TRAJ16 |  | LMNB1 |  |
| TRAJ17 |  | NMB |  |
| TRAJ18 |  | BNIP3 |  |
| TRAJ19 |  | RHOBTB3 |  |
| TRAJ20 |  | NCAPG |  |
| TRAJ21 |  | UBASH3A |  |
| TRAJ22 |  | ADAMTS10 |  |
| TRAJ23 |  | SEMA6B |  |
| TRAJ24 |  | NCMAP |  |
| TRAJ25 |  | AUH |  |
| TRAJ26 |  | HLA-DPA1 |  |
| TRAJ27 |  | CEACAM21 |  |
| TRAJ28 |  | LINGO1 |  |
| TRAJ29 |  | VDR |  |
| TRAJ30 |  | SLC41A2 |  |
| TRAJ31 |  | MAPK8IP3 |  |
| TRAJ32 |  | BTN3A3 |  |
| TRAJ33 |  | NFKB2 |  |
| TRAJ34 |  | VEPH1 |  |
| TRAJ35 |  | HDAC11 |  |
| TRAJ36 |  | ITK |  |
| TRAJ37 |  | PGGHG |  |
| TRAJ38 |  | PLEKHO2 |  |
| TRAJ39 |  | TUBB6 |  |
| TRAJ40 |  | SLC9A9 |  |
| TRAJ41 |  | MYO5C |  |
| TRAJ42 |  | MYOZ1 |  |
| TRAJ43 |  | THBS3 |  |
| TRAJ44 |  | CDS1 |  |
| TRAJ45 |  | MMP14 |  |
| TRAJ46 |  | ASS1 |  |
| TRAJ47 |  | CYBA |  |
| TRAJ48 |  | DOCK10 |  |
| TRAJ49 |  | CTHRC1 |  |
| TRAJ50 |  | HLA-DRA |  |
| TRAJ52 |  | COL5A2 |  |
| TRAJ53 |  | ADGRE1 |  |
| TRAJ54 |  | RIMKLA |  |
| TRAJ56 |  | PIMREG |  |
| TRAJ57 |  | ISG15 |  |
| TRAJ58 |  | CSF1R |  |
| TRAJ59 |  | ADAMTSL4 |  |
| TRAJ61 |  | TBX15 |  |
| TRAV1-1 |  | P4HA2 |  |
| TRAV1-2 |  | LY96 |  |
| TRAV2 |  | CA2 |  |
| TRAV3 |  | HHLA2 |  |
| TRAV4 |  | STK32A |  |
| TRAV5 |  | USP2 |  |
| TRAV7 |  | COL8A1 |  |
| TRAV8-1 |  | ADAMTS2 |  |
| TRAV8-2 |  | SLC25A4 |  |
| TRAV8-3 |  | IDH2 |  |
| TRAV8-4 |  | PCDHB10 |  |
| TRAV8-6 |  | SCARF1 |  |
| TRAV8-7 |  | TRAM1L1 |  |
| TRAV9-1 |  | NAPEPLD |  |
| TRAV9-2 |  | UNC5B |  |
| TRAV10 |  | SLC43A1 |  |
| TRAV12-1 |  | SIRPA |  |
| TRAV12-2 |  | AVPR1B |  |
| TRAV12-3 |  | QRFPR |  |
| TRAV13-1 |  | DNAJC11 |  |
| TRAV13-2 |  | ANKZF1 |  |
| TRAV14DV4 |  | FAS |  |
| TRAV16 |  | PDE7A |  |
| TRAV17 |  | MFNG |  |
| TRAV18 |  | DIPK2B |  |
| TRAV19 |  | RNASE2 |  |
| TRAV20 |  | PLK1 |  |
| TRAV21 |  | VAMP5 |  |
| TRAV22 |  | TFAP2C |  |
| TRAV23DV6 |  | MRPS6 |  |
| TRAV24 |  | P2RY8 |  |
| TRAV25 |  | FOXI2 |  |
| TRAV26-1 |  | DNAJC6 |  |
| TRAV26-2 |  | MYO6 |  |
| TRAV27 |  | PTGR1 |  |
| TRAV29DV5 |  | MYL5 |  |
| TRAV30 |  | CBLN3 |  |
| TRAV34 |  | VAV3 |  |
| TRAV35 |  | C5 |  |
| TRAV36DV7 |  | ETNPPL |  |
| TRAV38-1 |  | ARHGEF6 |  |
| TRAV38-2DV8 |  | TOX2 |  |
| TRAV39 |  | CYS1 |  |
| TRAV40 |  | L2HGDH |  |
| TRAV41 |  | VWA8 |  |
| TRBC1 |  | AGR3 |  |
| TRBC2 |  | SBNO2 |  |
| TRBD1 |  | DEGS1 |  |
| TRBD2 |  | FAM124A |  |
| TRBJ1-1 |  | SYPL2 |  |
| TRBJ1-2 |  | GRAMD4 |  |
| TRBJ1-3 |  | NLGN1 |  |
| TRBJ1-4 |  | NHSL1 |  |
| TRBJ1-5 |  | PNPLA1 |  |
| TRBJ1-6 |  | CAMK1D |  |
| TRBJ2-1 |  | PRSS8 |  |
| TRBJ2-2 |  | KIF18B |  |
| TRBJ2-3 |  | CLMN |  |
| TRBJ2-4 |  | HS3ST3B1 |  |
| TRBJ2-5 |  | PRKCQ |  |
| TRBJ2-6 |  | NEDD4L |  |
| TRBJ2-7 |  | KITLG |  |
| TRBV2 |  | UST |  |
| TRBV3-1 |  | SEMA3B |  |
| TRBV4-1 |  | TPSAB1 |  |
| TRBV4-2 |  | TNFSF14 |  |
| TRBV4-3 |  | QTRT1 |  |
| TRBV5-1 |  | HOXA11 |  |
| TRBV5-4 |  | TNIP1 |  |
| TRBV5-5 |  | HS3ST2 |  |
| TRBV5-6 |  | TMEM131L |  |
| TRBV5-7 |  | SOD3 |  |
| TRBV5-8 |  | LGI4 |  |
| TRBV6-1 |  | PTPN3 |  |
| TRBV6-2 |  | RTP4 |  |
| TRBV6-3 |  | EXOC3L4 |  |
| TRBV6-4 |  | TRIM14 |  |
| TRBV6-5 |  | PATL2 |  |
| TRBV6-6 |  | DARS1 |  |
| TRBV6-7 |  | COL1A1 |  |
| TRBV6-8 |  | DUSP10 |  |
| TRBV6-9 |  | FAM163A |  |
| TRBV7-2 |  | WT1 |  |
| TRBV7-3 |  | TBC1D9 |  |
| TRBV7-4 |  | PRRG1 |  |
| TRBV7-6 |  | BPHL |  |
| TRBV7-7 |  | FAM102A |  |
| TRBV7-8 |  | ACADM |  |
| TRBV7-9 |  | ADGRE2 |  |
| TRBV9 |  | GZMK |  |
| TRBV10-1 |  | PELI2 |  |
| TRBV10-2 |  | PXDN |  |
| TRBV10-3 |  | ENTPD8 |  |
| TRBV11-1 |  | PLOD1 |  |
| TRBV11-2 |  | WLS |  |
| TRBV11-3 |  | ABCA8 |  |
| TRBV12-3 |  | IFI44 |  |
| TRBV12-4 |  | PROC |  |
| TRBV12-5 |  | GPR183 |  |
| TRBV13 |  | SLC19A2 |  |
| TRBV14 |  | CORO1C |  |
| TRBV15 |  | POPDC2 |  |
| TRBV16 |  | IL1RL1 |  |
| TRBV17 |  | CD300LB |  |
| TRBV18 |  | HCST |  |
| TRBV19 |  | SIRPB1 |  |
| TRBV20-1 |  | C11orf21 |  |
| TRBV24-1 |  | FBLN5 |  |
| TRBV25-1 |  | ISM2 |  |
| TRBV27 |  | CXorf21 |  |
| TRBV28 |  | INF2 |  |
| TRBV29-1 |  | GLIPR1 |  |
| TRBV30 |  | MUC1 |  |
| TRDC |  | CAT |  |
| TRDD1 |  | FREM2 |  |
| TRDD2 |  | PCSK1N |  |
| TRDD3 |  | CP |  |
| TRDJ1 |  | ALDH4A1 |  |
| TRDJ2 |  | IDUA |  |
| TRDJ3 |  | SLC34A3 |  |
| TRDJ4 |  | HSD17B7 |  |
| TRDV1 |  | PCK2 |  |
| TRDV2 |  | HIBADH |  |
| TRDV3 |  | BTN2A2 |  |
| TRGV9 |  | SHROOM1 |  |
| TRGV8 |  | CDKL2 |  |
| TRGV5 |  | NDRG2 |  |
| TRGV4 |  | RBM47 |  |
| TRGV3 |  | SEMA4A |  |
| TRGV2 |  | NMI |  |
| TRGJP2 |  | C5orf46 |  |
| TRGJP1 |  | ANGPTL3 |  |
| TRGJP |  | MS4A4E |  |
| TRGJ2 |  | CNTNAP1 |  |
| TRGJ1 |  | TIGIT |  |
| TRGC2 |  | FHOD3 |  |
| TRGC1 |  | MCTP2 |  |
| TRAV6 |  | MYO15B |  |
|  |  | CHST13 |  |
|  |  | RARA |  |
|  |  | BTBD11 |  |
|  |  | ATP5F1B |  |
|  |  | IL2RG |  |
|  |  | SEL1L3 |  |
|  |  | TRIM22 |  |
|  |  | ZNF83 |  |
|  |  | BCAS1 |  |
|  |  | ELOVL2 |  |
|  |  | ANLN |  |
|  |  | OSMR |  |
|  |  | PLA2G4A |  |
|  |  | ABCB1 |  |
|  |  | TWSG1 |  |
|  |  | FMNL3 |  |
|  |  | SUCNR1 |  |
|  |  | ACAN |  |
|  |  | BMP6 |  |
|  |  | MAP3K12 |  |
|  |  | ABI3 |  |
|  |  | FCGR2B |  |
|  |  | PI16 |  |
|  |  | MTTP |  |
|  |  | CHODL |  |
|  |  | TM7SF2 |  |
|  |  | MAP3K14 |  |
|  |  | C4B |  |
|  |  | SACS |  |
|  |  | HEY1 |  |
|  |  | CREB5 |  |
|  |  | SDR42E1 |  |
|  |  | ITM2C |  |
|  |  | GFI1 |  |
|  |  | GLIPR2 |  |
|  |  | HAVCR2 |  |
|  |  | PLCG2 |  |
|  |  | PSCA |  |
|  |  | CD248 |  |
|  |  | B3GNT8 |  |
|  |  | CARD11 |  |
|  |  | TMEM38A |  |
|  |  | ARHGAP33 |  |
|  |  | KIT |  |
|  |  | SYNM |  |
|  |  | ACSM3 |  |
|  |  | KCNMA1 |  |
|  |  | APOL2 |  |
|  |  | CDCA5 |  |
|  |  | PPP1R3B |  |
|  |  | CDH6 |  |
|  |  | DAO |  |
|  |  | LRBA |  |
|  |  | WDR72 |  |
|  |  | PRKCA |  |
|  |  | CCRL2 |  |
|  |  | PCDH17 |  |
|  |  | FAM107A |  |
|  |  | RGS18 |  |
|  |  | S100A10 |  |
|  |  | EPS8L1 |  |
|  |  | SH3BGRL3 |  |
|  |  | WDR90 |  |
|  |  | BMERB1 |  |
|  |  | TAP2 |  |
|  |  | CENPF |  |
|  |  | ALDOC |  |
|  |  | ST3GAL4 |  |
|  |  | COL4A2 |  |
|  |  | PFN2 |  |
|  |  | MARCKS |  |
|  |  | FSCN1 |  |
|  |  | MCCC1 |  |
|  |  | P2RY13 |  |
|  |  | PIEZO2 |  |
|  |  | CDKN1C |  |
|  |  | ECRG4 |  |
|  |  | CLASRP |  |
|  |  | CENPA |  |
|  |  | SIGLEC7 |  |
|  |  | GIMAP4 |  |
|  |  | PRR36 |  |
|  |  | SLC43A2 |  |
|  |  | CCL18 |  |
|  |  | TMEM155 |  |
|  |  | MT1F |  |
|  |  | CALHM6 |  |
|  |  | SYTL4 |  |
|  |  | PLEKHG4 |  |
|  |  | GLIS1 |  |
|  |  | NTN4 |  |
|  |  | PLOD2 |  |
|  |  | TMEM255B |  |
|  |  | CDH1 |  |
|  |  | NCS1 |  |
|  |  | LYNX1 |  |
|  |  | YEATS2 |  |
|  |  | CYP26B1 |  |
|  |  | PIDD1 |  |
|  |  | CENPT |  |
|  |  | ST3GAL6 |  |
|  |  | ASPM |  |
|  |  | OASL |  |
|  |  | AFAP1L1 |  |
|  |  | NFKBID |  |
|  |  | AKIRIN1 |  |
|  |  | PARP15 |  |
|  |  | PBX1 |  |
|  |  | SLC28A1 |  |
|  |  | NAP1L3 |  |
|  |  | PMEPA1 |  |
|  |  | ANKRD37 |  |
|  |  | RASSF9 |  |
|  |  | NLRP2 |  |
|  |  | RGS5 |  |
|  |  | PRSS3 |  |
|  |  | NRM |  |
|  |  | SLAMF8 |  |
|  |  | AGER |  |
|  |  | P2RY10 |  |
|  |  | ADGRE5 |  |
|  |  | AGBL3 |  |
|  |  | ST14 |  |
|  |  | MTMR11 |  |
|  |  | HTRA4 |  |
|  |  | APCDD1L |  |
|  |  | MAP9 |  |
|  |  | NPDC1 |  |
|  |  | NEBL |  |
|  |  | TREH |  |
|  |  | COL27A1 |  |
|  |  | OR2A4 |  |
|  |  | GOLGA7B |  |
|  |  | EXOC3L1 |  |
|  |  | CD36 |  |
|  |  | LOXL3 |  |
|  |  | LAG3 |  |
|  |  | GPX2 |  |
|  |  | ALOX15B |  |
|  |  | TMEM25 |  |
|  |  | CKMT1B |  |
|  |  | LCAT |  |
|  |  | GJA1 |  |
|  |  | RFX2 |  |
|  |  | CLEC5A |  |
|  |  | NRBP2 |  |
|  |  | TRAT1 |  |
|  |  | ARL4A |  |
|  |  | CSF2RB |  |
|  |  | ICOS |  |
|  |  | APBB3 |  |
|  |  | MYBL1 |  |
|  |  | PLG |  |
|  |  | H1-0 |  |
|  |  | TLR7 |  |
|  |  | TUBA1A |  |
|  |  | LMO7 |  |
|  |  | CDCA8 |  |
|  |  | PANK1 |  |
|  |  | OR2I1P |  |
|  |  | CRYGS |  |
|  |  | PRLR |  |
|  |  | SLC22A13 |  |
|  |  | KCNJ2 |  |
|  |  | CENATAC |  |
|  |  | GRK5 |  |
|  |  | PLXNC1 |  |
|  |  | ACAT1 |  |
|  |  | CERKL |  |
|  |  | KLF5 |  |
|  |  | PLVAP |  |
|  |  | ALPK2 |  |
|  |  | CETP |  |
|  |  | KCNIP1 |  |
|  |  | SLC6A8 |  |
|  |  | CR589904.2 |  |
|  |  | IDNK |  |
|  |  | SHC1 |  |
|  |  | ITGAD |  |
|  |  | VWA5A |  |
|  |  | ADAMDEC1 |  |
|  |  | SDC3 |  |
|  |  | NGF |  |
|  |  | KCNK9 |  |
|  |  | LYZ |  |
|  |  | TRAF3IP3 |  |
|  |  | SLC10A6 |  |
|  |  | HS6ST3 |  |
|  |  | CD8B |  |
|  |  | C5AR1 |  |
|  |  | ANO1 |  |
|  |  | MAPK11 |  |
|  |  | SNX10 |  |
|  |  | NEK2 |  |
|  |  | SEMA3G |  |
|  |  | NLRP1 |  |
|  |  | FAHD2B |  |
|  |  | GALNT17 |  |
|  |  | CDC20 |  |
|  |  | PIK3CG |  |
|  |  | PTPN7 |  |
|  |  | SPATA18 |  |
|  |  | GYPC |  |
|  |  | LMTK3 |  |
|  |  | TPSB2 |  |
|  |  | FBXO21 |  |
|  |  | SH2B3 |  |
|  |  | CCL4L2 |  |
|  |  | PRR16 |  |
|  |  | NBPF15 |  |
|  |  | C3orf70 |  |
|  |  | MSX1 |  |
|  |  | GPHN |  |
|  |  | RAD54L |  |
|  |  | FBLN1 |  |
|  |  | PFKM |  |
|  |  | GIT2 |  |
|  |  | SVEP1 |  |
|  |  | GIMAP2 |  |
|  |  | C4A |  |
|  |  | LMNTD2 |  |
|  |  | ARHGAP42 |  |
|  |  | SLC39A14 |  |
|  |  | DCDC2 |  |
|  |  | C2 |  |
|  |  | GPR176 |  |
|  |  | CDCA7L |  |
|  |  | SYNGR1 |  |
|  |  | TLCD4 |  |
|  |  | MYEOV |  |
|  |  | KSR1 |  |
|  |  | ETNK2 |  |
|  |  | MYO10 |  |
|  |  | ZNRF3 |  |
|  |  | C9orf24 |  |
|  |  | BAIAP3 |  |
|  |  | RDH5 |  |
|  |  | RCSD1 |  |
|  |  | TNFRSF9 |  |
|  |  | ADCY7 |  |
|  |  | CADM1 |  |
|  |  | IGSF3 |  |
|  |  | ANG |  |
|  |  | MMUT |  |
|  |  | GPRC5B |  |
|  |  | OR51E2 |  |
|  |  | NRARP |  |
|  |  | STING1 |  |
|  |  | CIART |  |
|  |  | ANK3 |  |
|  |  | TLR8 |  |
|  |  | HOXD3 |  |
|  |  | MACROD1 |  |
|  |  | SMOX |  |
|  |  | CPNE5 |  |
|  |  | GABRE |  |
|  |  | G6PC |  |
|  |  | CYTIP |  |
|  |  | ADAM19 |  |
|  |  | SH3PXD2B |  |
|  |  | GBP1 |  |
|  |  | CEBPB |  |
|  |  | GOT1 |  |
|  |  | ATP2B2 |  |
|  |  | CCDC102B |  |
|  |  | NFE2L3 |  |
|  |  | ADRA2C |  |
|  |  | DYSF |  |
|  |  | FLI1 |  |
|  |  | ATP8B3 |  |
|  |  | AIM2 |  |
|  |  | CDK5RAP3 |  |
|  |  | RGCC |  |
|  |  | CREB3L3 |  |
|  |  | SLCO2B1 |  |
|  |  | GRB7 |  |
|  |  | RAB15 |  |
|  |  | FAM43B |  |
|  |  | DCLK1 |  |
|  |  | CTLA4 |  |
|  |  | SLC17A4 |  |
|  |  | LRRC75B |  |
|  |  | PDCD1 |  |
|  |  | FUT3 |  |
|  |  | CCR7 |  |
|  |  | ICAM1 |  |
|  |  | PLAUR |  |
|  |  | ACTG2 |  |
|  |  | HIBCH |  |
|  |  | VMP1 |  |
|  |  | CDCA7 |  |
|  |  | CCDC74A |  |
|  |  | ACLY |  |
|  |  | CLCN5 |  |
|  |  | CYP3A5 |  |
|  |  | FMO4 |  |
|  |  | OAS2 |  |
|  |  | RERGL |  |
|  |  | EPHX2 |  |
|  |  | KCNJ16 |  |
|  |  | LPIN3 |  |
|  |  | ADAM28 |  |
|  |  | XAF1 |  |
|  |  | UBXN11 |  |
|  |  | TUBB2A |  |
|  |  | FYB1 |  |
|  |  | PIGR |  |
|  |  | F2RL3 |  |
|  |  | ATP8B1 |  |
|  |  | COBL |  |
|  |  | WDFY4 |  |
|  |  | VWCE |  |
|  |  | ADRA1B |  |
|  |  | PTHLH |  |
|  |  | SH2D4A |  |
|  |  | MELTF |  |
|  |  | GPR174 |  |
|  |  | PLLP |  |
|  |  | PCK1 |  |
|  |  | INSR |  |
|  |  | VKORC1 |  |
|  |  | CCNL2 |  |
|  |  | BIRC7 |  |
|  |  | TGFBI |  |
|  |  | XCL1 |  |
|  |  | MMP9 |  |
|  |  | CHST15 |  |
|  |  | GPR34 |  |
|  |  | SLC2A5 |  |
|  |  | P4HA3 |  |
|  |  | ALDH5A1 |  |
|  |  | GUCY1B1 |  |
|  |  | THEMIS |  |
|  |  | ASPDH |  |
|  |  | IGF2BP2 |  |
|  |  | C14orf132 |  |
|  |  | PGM5 |  |
|  |  | PLAC8 |  |
|  |  | HIGD1B |  |
|  |  | CAPN11 |  |
|  |  | GPR171 |  |
|  |  | LAMA2 |  |
|  |  | HEYL |  |
|  |  | NLRP3 |  |
|  |  | APOL6 |  |
|  |  | APOD |  |
|  |  | CAPN5 |  |
|  |  | TMEM273 |  |
|  |  | HYAL1 |  |
|  |  | EFNA1 |  |
|  |  | KCNJ11 |  |
|  |  | DOCK8 |  |
|  |  | FLT1 |  |
|  |  | PRRT2 |  |
|  |  | PHYH |  |
|  |  | POGLUT2 |  |
|  |  | CDON |  |
|  |  | BDKRB2 |  |
|  |  | SOX11 |  |
|  |  | DSP |  |
|  |  | LAMC2 |  |
|  |  | TEX11 |  |
|  |  | RASL11A |  |
|  |  | MEF2C |  |
|  |  | DOCK6 |  |
|  |  | HOXD10 |  |
|  |  | PHGDH |  |
|  |  | SH3RF3 |  |
|  |  | TSPYL1 |  |
|  |  | FGFBP2 |  |
|  |  | TMEM245 |  |
|  |  | RAP1GAP2 |  |
|  |  | MARCHF1 |  |
|  |  | FILIP1 |  |
|  |  | NID1 |  |
|  |  | MET |  |
|  |  | GCAT |  |
|  |  | CCND2 |  |
|  |  | CPEB4 |  |
|  |  | FRY |  |
|  |  | PLA1A |  |
|  |  | ANO4 |  |
|  |  | TUBA1B |  |
|  |  | TNFRSF25 |  |
|  |  | IFI27 |  |
|  |  | GDF3 |  |
|  |  | LIPG |  |
|  |  | ADAMTSL2 |  |
|  |  | LBHD2 |  |
|  |  | CKMT2 |  |
|  |  | LTC4S |  |
|  |  | SLC17A2 |  |
|  |  | BST2 |  |
|  |  | CXCL13 |  |
|  |  | NFATC2 |  |
|  |  | SNAP25 |  |
|  |  | DLK2 |  |
|  |  | ADSS1 |  |
|  |  | TEK |  |
|  |  | TSPAN18 |  |
|  |  | TNFAIP2 |  |
|  |  | KCNMB1 |  |
|  |  | ECSCR |  |
|  |  | ACE |  |
|  |  | DHPS |  |
|  |  | C1QL1 |  |
|  |  | LAPTM4B |  |
|  |  | IRF9 |  |
|  |  | PTAFR |  |
|  |  | IRF1 |  |
|  |  | PAQR6 |  |
|  |  | DIAPH2 |  |
|  |  | FN1 |  |
|  |  | RGN |  |
|  |  | NKAIN1 |  |
|  |  | NDRG1 |  |
|  |  | SEMA3F |  |
|  |  | GLRB |  |
|  |  | SLC25A30 |  |
|  |  | CYBB |  |
|  |  | JUP |  |
|  |  | LARGE2 |  |
|  |  | CLMP |  |
|  |  | NPM2 |  |
|  |  | ACCS |  |
|  |  | SH3D21 |  |
|  |  | RIC3 |  |
|  |  | SCAMP5 |  |
|  |  | RCN3 |  |
|  |  | PIPOX |  |
|  |  | SCNN1D |  |
|  |  | ASAP1 |  |
|  |  | PAH |  |
|  |  | RIT1 |  |
|  |  | FSTL3 |  |
|  |  | RFTN1 |  |
|  |  | LCN2 |  |
|  |  | FGFR2 |  |
|  |  | WDR54 |  |
|  |  | HLA-DOB |  |
|  |  | PLS3 |  |
|  |  | FSTL1 |  |
|  |  | AIF1 |  |
|  |  | ABCA12 |  |
|  |  | MPEG1 |  |
|  |  | DDX39B |  |
|  |  | LIX1 |  |
|  |  | SIPA1L2 |  |
|  |  | NGFR |  |
|  |  | HLA-DMB |  |
|  |  | C16orf74 |  |
|  |  | SYNE2 |  |
|  |  | LY6E |  |
|  |  | PDLIM7 |  |
|  |  | GYG2 |  |
|  |  | GJB1 |  |
|  |  | CPVL |  |
|  |  | SCGN |  |
|  |  | JPH2 |  |
|  |  | CTF1 |  |
|  |  | MFHAS1 |  |
|  |  | KCNK3 |  |
|  |  | FRMD1 |  |
|  |  | WNT11 |  |
|  |  | S100A8 |  |
|  |  | TMEM200A |  |
|  |  | NTN1 |  |
|  |  | IL6R |  |
|  |  | EVA1C |  |
|  |  | CPA3 |  |
|  |  | EYA2 |  |
|  |  | RRAD |  |
|  |  | ACOX2 |  |
|  |  | LPAR6 |  |
|  |  | MARVELD1 |  |
|  |  | ZP3 |  |
|  |  | ENPP1 |  |
|  |  | CDH4 |  |
|  |  | SPON2 |  |
|  |  | CD44 |  |
|  |  | MCHR1 |  |
|  |  | PPL |  |
|  |  | PRKD1 |  |
|  |  | ADH6 |  |
|  |  | SEPTIN4 |  |
|  |  | NFASC |  |
|  |  | PCDHB14 |  |
|  |  | CD200R1 |  |
|  |  | ARL4C |  |
|  |  | CST6 |  |
|  |  | SCN4B |  |
|  |  | RNF182 |  |
|  |  | H3C10 |  |
|  |  | FRAS1 |  |
|  |  | WNT10A |  |
|  |  | CYP4F3 |  |
|  |  | EME2 |  |
|  |  | RASL12 |  |
|  |  | RHEX |  |
|  |  | SLC16A6 |  |
|  |  | NOTCH4 |  |
|  |  | SIGLEC14 |  |
|  |  | MAF |  |
|  |  | CD69 |  |
|  |  | SLPI |  |
|  |  | EGFR |  |
|  |  | PLEKHA6 |  |
|  |  | GPR155 |  |
|  |  | PDGFD |  |
|  |  | MYO5B |  |
|  |  | IL7R |  |
|  |  | LCK |  |
|  |  | HIF1A |  |
|  |  | C1orf210 |  |
|  |  | SLC16A11 |  |
|  |  | APOLD1 |  |
|  |  | NRP1 |  |
|  |  | IRAG2 |  |
|  |  | CEACAM1 |  |
|  |  | C6orf132 |  |
|  |  | GPT2 |  |
|  |  | CCDC85B |  |
|  |  | SCG2 |  |
|  |  | IRS1 |  |
|  |  | PROM1 |  |
|  |  | MALL |  |
|  |  | PROS1 |  |
|  |  | SORCS2 |  |
|  |  | LDLR |  |
|  |  | TLR3 |  |
|  |  | BHLHA15 |  |
|  |  | APOL1 |  |
|  |  | QPRT |  |
|  |  | FAM83F |  |
|  |  | F3 |  |
|  |  | CLK1 |  |
|  |  | CYP1B1 |  |
|  |  | AHCYL2 |  |
|  |  | IL20RB |  |
|  |  | NPY1R |  |
|  |  | FOLR2 |  |
|  |  | KCNAB1 |  |
|  |  | RELN |  |
|  |  | DOC2B |  |
|  |  | TMEM92 |  |
|  |  | LGR4 |  |
|  |  | SSPN |  |
|  |  | ASAP2 |  |
|  |  | INKA1 |  |
|  |  | MASP1 |  |
|  |  | PCDH1 |  |
|  |  | APOC3 |  |
|  |  | ALDH1B1 |  |
|  |  | CPN2 |  |
|  |  | SMIM10 |  |
|  |  | PLPPR5 |  |
|  |  | TDRD9 |  |
|  |  | TST |  |
|  |  | RAB29 |  |
|  |  | PABPC1L |  |
|  |  | ANGPTL2 |  |
|  |  | FOXJ3 |  |
|  |  | PLAAT4 |  |
|  |  | SERPINE1 |  |
|  |  | CLEC1A |  |
|  |  | MAMDC4 |  |
|  |  | PC |  |
|  |  | SPARCL1 |  |
|  |  | WFDC2 |  |
|  |  | CA4 |  |
|  |  | CCL20 |  |
|  |  | COL5A1 |  |
|  |  | PER2 |  |
|  |  | RARRES2 |  |
|  |  | CD82 |  |
|  |  | SOWAHB |  |
|  |  | VWA1 |  |
|  |  | CEBPA |  |
|  |  | RDH12 |  |
|  |  | CD163 |  |
|  |  | FBLN7 |  |
|  |  | CIITA |  |
|  |  | FPR1 |  |
|  |  | TOX |  |
|  |  | REC8 |  |
|  |  | DACT2 |  |
|  |  | VCAN |  |
|  |  | MEST |  |
|  |  | ADAMTSL1 |  |
|  |  | KCNN1 |  |
|  |  | LRRC17 |  |
|  |  | EPHA3 |  |
|  |  | COL6A1 |  |
|  |  | TLN2 |  |
|  |  | VSIG4 |  |
|  |  | LAYN |  |
|  |  | TGM2 |  |
|  |  | LRATD2 |  |
|  |  | TRIB1 |  |
|  |  | UGT3A1 |  |
|  |  | RNF152 |  |
|  |  | MATN2 |  |
|  |  | BTNL9 |  |
|  |  | RFLNB |  |
|  |  | SPHK1 |  |
|  |  | LENG8 |  |
|  |  | GIMAP7 |  |
|  |  | PDZRN3 |  |
|  |  | SPINK13 |  |
|  |  | GSDMB |  |
|  |  | HTR6 |  |
|  |  | CHDH |  |
|  |  | CFD |  |
|  |  | FZD1 |  |
|  |  | COL21A1 |  |
|  |  | POSTN |  |
|  |  | IFI44L |  |
|  |  | FBXO41 |  |
|  |  | CNTFR |  |
|  |  | CLRN3 |  |
|  |  | GLB1L |  |
|  |  | CCR2 |  |
|  |  | VCAM1 |  |
|  |  | SCTR |  |
|  |  | SPRY4 |  |
|  |  | CLDN7 |  |
|  |  | FOXF1 |  |
|  |  | IRX3 |  |
|  |  | PKHD1 |  |
|  |  | CDHR1 |  |
|  |  | CRYL1 |  |
|  |  | CCN6 |  |
|  |  | TMEM200B |  |
|  |  | NCOA7 |  |
|  |  | MUC13 |  |
|  |  | PREX2 |  |
|  |  | SLIT2 |  |
|  |  | CYP24A1 |  |
|  |  | CCNO |  |
|  |  | GUCA2B |  |
|  |  | BCO1 |  |
|  |  | RAB11FIP1 |  |
|  |  | HECW2 |  |
|  |  | COL15A1 |  |
|  |  | CKB |  |
|  |  | GOLGA8A |  |
|  |  | APOH |  |
|  |  | CXCL12 |  |
|  |  | TSPYL2 |  |
|  |  | TCF19 |  |
|  |  | CX3CR1 |  |
|  |  | FLRT3 |  |
|  |  | TTLL3 |  |
|  |  | PLAAT2 |  |
|  |  | OAS3 |  |
|  |  | H2AC13 |  |
|  |  | SELE |  |
|  |  | SCRN1 |  |
|  |  | PARM1 |  |
|  |  | SPRY1 |  |
|  |  | CLIC3 |  |
|  |  | ATP9A |  |
|  |  | SPINK1 |  |
|  |  | SORL1 |  |
|  |  | DNAJB13 |  |
|  |  | LIPA |  |
|  |  | EVC |  |
|  |  | NRXN2 |  |
|  |  | SHANK3 |  |
|  |  | CSAD |  |
|  |  | FTH1 |  |
|  |  | PKIA |  |
|  |  | ALOX5AP |  |
|  |  | AQP7 |  |
|  |  | IFNG |  |
|  |  | SMTNL2 |  |
|  |  | ADM2 |  |
|  |  | C10orf99 |  |
|  |  | SPSB1 |  |
|  |  | CCDC8 |  |
|  |  | B3GNT4 |  |
|  |  | P2RY12 |  |
|  |  | APLNR |  |
|  |  | RNF180 |  |
|  |  | AJAP1 |  |
|  |  | PROSER2 |  |
|  |  | HAVCR1 |  |
|  |  | SHC2 |  |
|  |  | IL2RA |  |
|  |  | SELP |  |
|  |  | LGALS12 |  |
|  |  | VNN2 |  |
|  |  | UNC5D |  |
|  |  | MYL3 |  |
|  |  | GJA4 |  |
|  |  | GIPC3 |  |
|  |  | DDAH1 |  |
|  |  | COL1A2 |  |
|  |  | SDC1 |  |
|  |  | CCN2 |  |
|  |  | TRIM50 |  |
|  |  | LUM |  |
|  |  | TP53I11 |  |
|  |  | KL |  |
|  |  | PILRB |  |
|  |  | ARC |  |
|  |  | IL22RA1 |  |
|  |  | FHL1 |  |
|  |  | SLC4A3 |  |
|  |  | ATP11A |  |
|  |  | TSPAN1 |  |
|  |  | GPR35 |  |
|  |  | GDF15 |  |
|  |  | TTR |  |
|  |  | C19orf67 |  |
|  |  | PECAM1 |  |
|  |  | CFAP126 |  |
|  |  | UPK1B |  |
|  |  | SYNPO |  |
|  |  | ADGRA2 |  |
|  |  | CSDC2 |  |
|  |  | FOXS1 |  |
|  |  | EDNRA |  |
|  |  | CAPN6 |  |
|  |  | MPZL2 |  |
|  |  | ITPKA |  |
|  |  | BEX2 |  |
|  |  | HLA-DRB5 |  |
|  |  | LY6H |  |
|  |  | OSM |  |
|  |  | DNAJC22 |  |
|  |  | CLEC18A |  |
|  |  | CIB4 |  |
|  |  | TIMD4 |  |
|  |  | ETS1 |  |
|  |  | HEY2 |  |
|  |  | EGFLAM |  |
|  |  | TNFRSF11B |  |
|  |  | PTGES |  |
|  |  | METTL7B |  |
|  |  | TBC1D14 |  |
|  |  | ENPEP |  |
|  |  | KCNJ15 |  |
|  |  | PODXL2 |  |
|  |  | GOLGA8B |  |
|  |  | NAPSA |  |
|  |  | GOLM1 |  |
|  |  | KLHDC7A |  |
|  |  | BEX1 |  |
|  |  | STUM |  |
|  |  | EDN1 |  |
|  |  | PADI1 |  |
|  |  | SOD2 |  |
|  |  | NOTCH3 |  |
|  |  | TPPP3 |  |
|  |  | FCN3 |  |
|  |  | CXADR |  |
|  |  | TGFA |  |
|  |  | TUBA3D |  |
|  |  | CDH5 |  |
|  |  | MAB21L3 |  |
|  |  | VANGL2 |  |
|  |  | PGBD5 |  |
|  |  | SLC22A4 |  |
|  |  | ARHGAP5 |  |
|  |  | BCL6B |  |
|  |  | RHOBTB1 |  |
|  |  | FGF7 |  |
|  |  | SCGB1D2 |  |
|  |  | ENTPD2 |  |
|  |  | THRSP |  |
|  |  | BEX5 |  |
|  |  | RASSF6 |  |
|  |  | CHI3L2 |  |
|  |  | ANK1 |  |
|  |  | CLEC14A |  |
|  |  | CYTL1 |  |
|  |  | FGL2 |  |
|  |  | PAIP2B |  |
|  |  | OGN |  |
|  |  | SCGB3A2 |  |
|  |  | EMCN |  |
|  |  | MAPK15 |  |
|  |  | CES3 |  |
|  |  | TRIM15 |  |
|  |  | SPNS2 |  |
|  |  | GPC4 |  |
|  |  | SLC22A6 |  |
|  |  | BCHE |  |
|  |  | STAC2 |  |
|  |  | HLA-DQA2 |  |
|  |  | DMKN |  |
|  |  | MOGAT3 |  |
|  |  | ADORA1 |  |
|  |  | TREX2 |  |
|  |  | SLC16A12 |  |
|  |  | PBLD |  |
|  |  | ELF3 |  |
|  |  | CCL28 |  |
|  |  | FOLH1 |  |
|  |  | HSD11B1 |  |
|  |  | TRABD2B |  |
|  |  | GPM6A |  |
|  |  | TMEM176A |  |
|  |  | CDH2 |  |
|  |  | ENPP5 |  |
|  |  | CCR1 |  |
|  |  | COL14A1 |  |
|  |  | ACSM5 |  |
|  |  | TMEM125 |  |
|  |  | PCDHGC3 |  |
|  |  | MEI4 |  |
|  |  | TMEM174 |  |
|  |  | AQP9 |  |
|  |  | GATM |  |
|  |  | TNMD |  |
|  |  | FLT4 |  |
|  |  | PTGS2 |  |
|  |  | PODXL |  |
|  |  | FOXQ1 |  |
|  |  | PRND |  |
|  |  | AKR7A3 |  |
|  |  | BAIAP2L2 |  |
|  |  | DPYSL3 |  |
|  |  | CDH13 |  |
|  |  | CLDN4 |  |
|  |  | TSC22D3 |  |
|  |  | SULF1 |  |
|  |  | GASK1B |  |
|  |  | PLA2G2D |  |
|  |  | USH1C |  |
|  |  | MT1M |  |
|  |  | CHIT1 |  |
|  |  | MAOA |  |
|  |  | COL3A1 |  |
|  |  | MXRA5 |  |
|  |  | HAO2 |  |
|  |  | CLEC18B |  |
|  |  | SLC22A3 |  |
|  |  | OLR1 |  |
|  |  | FOLR1 |  |
|  |  | AGT |  |
|  |  | PRELP |  |
|  |  | ZNF385B |  |
|  |  | CRTAC1 |  |
|  |  | CD109 |  |
|  |  | FBXL16 |  |
|  |  | ACTA2 |  |
|  |  | STEAP4 |  |
|  |  | REG1A |  |
|  |  | C1QTNF1 |  |
|  |  | H2BC12 |  |
|  |  | CD5L |  |
|  |  | GBP4 |  |
|  |  | CTXN1 |  |
|  |  | SLC6A19 |  |
|  |  | S100A9 |  |
|  |  | RAMP1 |  |
|  |  | MFAP4 |  |
|  |  | HLA-DOA |  |
|  |  | CBLN4 |  |
|  |  | C1QL4 |  |
|  |  | MAP7D2 |  |
|  |  | SEMA3D |  |
|  |  | LTF |  |
|  |  | CRABP2 |  |
|  |  | SPX |  |
|  |  | CCL3L3 |  |
|  |  | CYP8B1 |  |
|  |  | AGMAT |  |
|  |  | B4GALNT1 |  |
|  |  | SLC17A3 |  |
|  |  | ANXA13 |  |
|  |  | SLC22A12 |  |
|  |  | MT1E |  |
|  |  | GXYLT2 |  |
|  |  | MGARP |  |
|  |  | FCGBP |  |
|  |  | PHYHIPL |  |
|  |  | ADAM12 |  |
|  |  | LRRN4 |  |
|  |  | PKP3 |  |
|  |  | MMP7 |  |
|  |  | MT1X |  |
|  |  | ABO |  |
|  |  | CLEC18C |  |
|  |  | MARCO |  |
|  |  | PRUNE2 |  |
|  |  | NTM |  |
|  |  | COL6A3 |  |
|  |  | GRIA4 |  |
|  |  | GJB2 |  |
|  |  | SPOCK1 |  |
|  |  | CDHR5 |  |
|  |  | DES |  |
|  |  | IGFBP5 |  |
|  |  | PF4V1 |  |
|  |  | AQP1 |  |
|  |  | G0S2 |  |
|  |  | KLF15 |  |
|  |  | CD79A |  |
|  |  | MDK |  |
|  |  | CXCL5 |  |
|  |  | CES1 |  |
|  |  | PRIMA1 |  |
|  |  | SMOC1 |  |
|  |  | SMIM32 |  |
|  |  | ALDH1L1 |  |
|  |  | FRZB |  |
|  |  | NR4A3 |  |
|  |  | ALDH8A1 |  |
|  |  | SERPINA1 |  |
|  |  | PPDPFL |  |
|  |  | CYP4A22 |  |
|  |  | ENPP2 |  |
|  |  | ATF3 |  |
|  |  | AOX1 |  |
|  |  | ALKAL2 |  |
|  |  | ACKR1 |  |
|  |  | CADM3 |  |
|  |  | EPO |  |
|  |  | IGLON5 |  |
|  |  | SORCS3 |  |
|  |  | SCARA3 |  |
|  |  | JCHAIN |  |
|  |  | PRAME |  |
|  |  | MUC20 |  |
|  |  | MAPT |  |
|  |  | POF1B |  |
|  |  | MZB1 |  |
|  |  | SMIM24 |  |
|  |  | CXCL2 |  |
|  |  | F2 |  |
|  |  | MT3 |  |
|  |  | RBP4 |  |
|  |  | NR4A1 |  |
|  |  | SERPINF2 |  |
|  |  | APOB |  |
|  |  | ISLR |  |
|  |  | GPR143 |  |
|  |  | LGALS2 |  |
|  |  | C1S |  |
|  |  | AZGP1 |  |
|  |  | SERPINA6 |  |
|  |  | CYP4A11 |  |
|  |  | ERAP2 |  |
|  |  | EGR1 |  |
|  |  | FCAMR |  |
|  |  | FGG |  |
|  |  | MIOX |  |
|  |  | SLC5A1 |  |
|  |  | SLC13A1 |  |
|  |  | GSG1L2 |  |
|  |  | TMEM252 |  |
|  |  | KRT19 |  |
|  |  | CCL21 |  |
|  |  | LGALS4 |  |
|  |  | SLC34A2 |  |
|  |  | SAA1 |  |
|  |  | AOC1 |  |
