# Supplementary material for: Integrative multi-omics analysis identifies a PTM-related immune signature and IRF9 as a driver in ccRCC
Source: Front Immunol. 2025 Dec 1;16:1707375. doi: 10.3389/fimmu.2025.1707375 (PMC12702869; doi:10.3389/fimmu.2025.1707375)
Supplement: Supplementary Figure 1 — Neural network model and predictive performance. (A) Model architecture after 100 epochs with accuracy of 0.98 and loss of 0.05. (B–D) Training set performance with nearly perfect classification (AUC = 1.000). (E–G) Testing set performance: confusion matrix, prediction distribution, and ROC curve (AUC = 0.942). (H–J) External validation in GSE40435 showing good predictive ability (AUC = 0.906) with reduced specificity. [file Supplementaryfile1.docx]

**
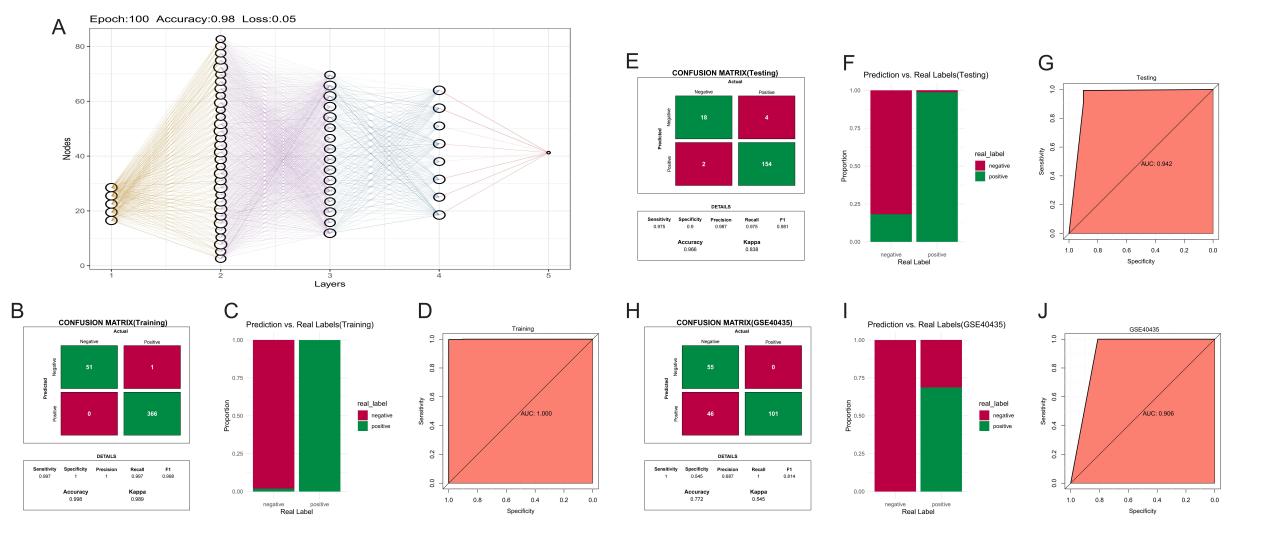
**

Figure S1. Neural network model and predictive performance.
(A) Model architecture after 100 epochs with accuracy of 0.98 and loss of 0.05. (B-D) Training set performance with nearly perfect classification (AUC = 1.000). (E-G) Testing set performance: confusion matrix, prediction distribution, and ROC curve (AUC = 0.942). (H-J) External validation in GSE40435 showing good predictive ability (AUC = 0.906) with reduced specificity.
